# Electrophysiological Properties of the Medial Mammillary Bodies across the Sleep–Wake Cycle

**DOI:** 10.1523/ENEURO.0447-23.2024

**Published:** 2024-04-24

**Authors:** Christopher M. Dillingham, Jonathan J. Wilson, Seralynne D. Vann

**Affiliations:** ^1^School of Psychology, Cardiff University, Cardiff CF10 3AT, United Kingdom; ^2^Neuroscience and Mental Health Innovation Institute, Cardiff CF24 4HQ, United Kingdom

## Abstract

The medial mammillary bodies (MBs) play an important role in the formation of spatial memories; their dense inputs from hippocampal and brainstem regions makes them well placed to integrate movement-related and spatial information, which is then extended to the anterior thalamic nuclei and beyond to the cortex. While the anatomical connectivity of the medial MBs has been well studied, much less is known about their physiological properties, particularly in freely moving animals. We therefore carried out a comprehensive characterization of medial MB electrophysiology across arousal states by concurrently recording from the medial MB and the CA1 field of the hippocampus in male rats. In agreement with previous studies, we found medial MB neurons to have firing rates modulated by running speed and angular head velocity, as well as theta-entrained firing. We extended the characterization of MB neuron electrophysiology in three key ways: (1) we identified a subset of neurons (25%) that exhibit dominant bursting activity; (2) we showed that ∼30% of theta-entrained neurons exhibit robust theta cycle skipping, a firing characteristic that implicates them in a network for prospective coding of position; and (3) a considerable proportion of medial MB units showed sharp-wave ripple (SWR) responsive firing (∼37%). The functional heterogeneity of MB electrophysiology reinforces their role as an integrative node for mnemonic processing and identifies potential roles for the MBs in memory consolidation through propagation of SWR-responsive activity to the anterior thalamus and prospective coding in the form of theta cycle skipping.

## Significance Statement

While the medial mammillary bodies (MBs) are important for memory, it is still not clear how they support memory formation. Through conjoint medial MB and hippocampal recordings across different arousal states, we identified a population of medial MB units with diverse and often conjunctive physiological properties, including theta-entrained cells, cells modulated by running speed and angular head velocity, complex bursting, theta cycle skipping activity, and hippocampal sharp-wave ripple-responsive firing. These properties likely support a role for the medial MBs in mnemonic processing, enabling the integration of separate sensory streams and the propagation of information to the thalamus.

## Introduction

The medial mammillary bodies (MBs) were one of the earliest regions associated with amnesia and play an important role in the formation of complex spatial memories, via their indirect influence on hippocampocortical networks (Vann and Albasser, 2009; Dillingham et al., 2015, 2019; Zakowski et al., 2017; McNaughton and Vann, 2022). Compared with our detailed knowledge of the anatomical connectivity of the medial MBs, our knowledge of the electrophysiological profiles of the medial MBs in behaving animals is relatively limited. While a few studies have been carried out using in vitro preparations (Alonso and Llinas, 1992), or in anesthetized and/or head-fixed animals (Kocsis and Vertes, 1994, 1997; Bland et al., 1995; Kirk et al., 1996), there is very little published data from awake, behaving rats (Sharp and Turner-Williams, 2005).

**Table 1.** Statistical table detailing the data structure, type of statistical test and the test statistic or power of each inferential statistic used

Ref	Figure	Data distribution	Test	Power/test statistic
a		Normal	Linear mixed effects	−1.361 ± 16.870
b	1*e*	Normal	Linear regression	*R*^2^ = 0.57
c				*R*^2^ = 0.52
d			Nonlinear curve fit—sigmoid function/BIC weighting	*R*^2^ = 0.90/Sigmoid BICw = 1
e				*R*^2^ = 0.88/Sigmoid BICw = 1
f	1*f*	Normal	Linear regression	*R*^2^ = 0.54
g				*R*^2^ = 0.45
h			Nonlinear curve fit—sigmoid function/BIC weighting	*R*^2^ = 0.67/Sigmoid BICw = 1
i				*R*^2^ = 0.90/Sigmoid BICw = 0.02
j	1*h*	Normal	General linear model	Diff: 0.48 ± 0.06
k	2*c*	Normal	Linear regression	Slope: 0.92 ± 0.01, *R*^2^ = 0.93
l	2*d*	Non-normal	Wilcoxon signed rank	*z* = 4.37
m				*z* = 4.35
n	2*f*	Circular	Rayleigh *Z*	*Z* = 0.47
o	3*c*	Normal	Linear mixed effects	Diff: −0.070 ± 0.003
p	3*d*	Normal	Linear mixed effects	Diff: −20.941 ± 1.125
q	3*e*,*f*	Normal	General linear model	6.13 ± 0.54
r	3*g*	Normal	Linear mixed effects	Diff: 0.14 ± 0.05
s	4*a*	Normal	General linear model	0.84 ± 0.17
t	4*b*	Circular	Circular correlation	*r* = 0.88
u	4*c*	Normal	Linear regression	Slope: 0.73 ± 0.04
v				Slope: 0.91 ± 0.05
w				Slope: 1.1 ± 0.06
x	4*e*	Circular	Rayleigh Z	*Z* = 57.75
y	4*g* (top)	Non-normal	Friedman	*χ*^2^ = 29.93
z			Dunn post hoc pairwise	Diff: −0.25 ± 0.18^a^
aa				Diff: −0.28 ± 0.21^a^
ab				Diff: 0.01 ± 0.08^a^
ac			Wilcoxon signed rank	−0.40 ± 0.13^a^
ad				−0.13 ± 0.23^a^
ae				−0.39 ± 0.20^a^
af			Dunn post hoc pairwise	Diff: 0.04 ± 0.29^a^
ag				Diff: 0.0. ± 0.29^a^
ah				Diff: 0.07 ± 0.10^a^
ai			Wilcoxon signed rank	0.03 ± 0.31^a^
aj				−0.17 ± 0.41^a^
ak				−0.05 ± 0.26^a^
al	4*g* (bottom)	Non-normal	Friedman	*χ*^2^ = 23.29
am			Dunn post hoc pairwise	Diff: 0.00 ± 0.05^a^
an				Diff: −0.01 ± 0.04^a^
ao				Diff: 0.01 ± 0.01^a^
ap				Diff: −0.04 ± 0.03^a^
aq				Diff: −0.02 ± 0.03^a^
ar				Diff: 0.00 ± 0.01^a^
as	5*f*	Normal	General linear model	Diff: 0.024 ± 0.01

a Differences for nonparametric tests are expressed as median ± IQR.

Letters in the “Ref” column refer to those denoted in superscript after each statistic in the main text.

From previous electrophysiological studies, we know that cells in the medial MBs show complex endogenous bursting activity (Alonso and Llinas, 1992; Kocsis and Vertes, 1994) and that a large proportion of neurons have spike discharges that are significantly phase entrained to theta band oscillations (Bland et al., 1995). Additionally, firing rates of medial MB units have been reported to be highly correlated with both running speed and angular head velocity (AHV; Sharp and Turner-Williams, 2005). While, historically, the medial MBs have been considered simply a relay of hippocampal theta, the emerging picture appears more complex, with evidence suggesting a bidirectional influence between the hippocampus (HPC) and medial MBs. Lesions (Sharp and Koester, 2008) or inactivation (Zakowski et al., 2017) of the MBs, or lesions of their primary efferent pathway, the mammillothalamic tract (Dillingham et al., 2019), alter the temporal dynamics of hippocampal processing, for example, through attenuation of theta band oscillatory frequency, highlighting the downstream impact of the MBs.

The present study sought to provide a comprehensive analysis of the electrophysiological properties of the medial MBs, extending beyond previous work by recording from greater numbers of neurons and, importantly, recording across different arousal states. A secondary goal was to assess the relationship between MB activity and hippocampal ripple band activity. The medial MBs receive a dense input from the subiculum, via the postcommissural fornix, and the MB-projecting neurons in the subiculum are strongly modulated by hippocampal sharp-wave ripple (SWR) events (Kitanishi et al., 2021). The prediction, therefore, is that MB activity will also be related to hippocampal SWR events. Given the strong links between the propagation of ripple activity and memory, this may be an additional mechanism via which the extended hippocampal–mammillary network supports long-term memory formation.

## Materials and Methods

### Ethics

All experimental procedures were in accordance with the UK Animals (Scientific Procedures) Act, 1986 and associated guidelines, the EU directive 2010/63/EU, as well as the Cardiff University Biological Standards Committee.

### Animals

Eight male Lister Hooded rats were used (Envigo), weighing ∼300–350 g at the time of surgery and behaviorally naive. Prior to surgery, rats were group housed. Following surgery, implanted rats were housed singly (GR1800 double-decker cages; Tecniplast) except for ∼1 h daily, supervised socialization. Animals were maintained under diurnal light conditions (14/10 h light/dark) with *ad libitum* access to water and environmental enrichment. When required, animals were food-deprived to no less than 85% of their *ad libitum* feeding weight to promote reward-based exploration.

### Electrode implantation

All surgeries were performed under an isoflurane–oxygen mixture (induction 5%, maintenance 2–2.5% isoflurane) during the light phase of a 12 h day/night cycle. Once anesthetized, animals were positioned in a stereotaxic frame (David Kopf Instruments). The skull was exposed and cleaned before 6–9 screws were secured and cemented to the skull. Craniotomies were made before careful removal of the dura and subsequent implantation of electrodes in positions corresponding to the following coordinates (mm from bregma unless stated): CA1: AP, −3.8; ML, ±3.3; DV, −1.9 from the top of the cortex 4.4; ML, ±0.8; DV, −8.0 from top of the cortex with a 4° angle toward the midline. Rats were implanted with 8 tetrodes (17 µm tungsten; California Fine Wire) into CA1, spanning ∼1.4 mm in the axial plane, and 4 octrodes (12 µm; California Fine Wire) into the MBs. Prior to implantation, electrodes were loaded into custom-made microdrives (Axona) that allow for independent adjustment of electrode depth during recordings. Electrodes were then gold-plated (Gold Plating Solution; NeuraLynx) to an impedance of ∼200–400 kΩ using the open-source Tetroplater (https://github.com/MatsumotoJ/Tetroplater). Once lowered to the desired depth, a warmed paraffin/mineral oil (1:4) suspension, or synthetic dura (Dura-Gel; Cambridge NeuroTech), was applied to the dural surface and allowed to solidify or set, respectively. Drives were then secured in place with bone cement (Zimmer Biomet). The scalp was then sutured around the drive. Postsurgery, animals were rehydrated with a subcutaneous 5–10 ml injection of 4% glucose saline and given postoperative analgesia (0.06 ml, 5 mg/ml meloxicam, Boehringer Ingelheim).

### Behavior

Recordings were performed in a custom-made circular track arena (diameter, 1.3 m) with a central sleep box (0.4 × 0.4 m). For awake (AWK) recordings, animals were trained to run outwardly around the track by accessing a small quantity of 50% water/condensed milk (by triggering one of two motion detectors positioned at 180° to one another) and returning to retrieve an experimenter-placed Cheerios (Nestle) reward at the start location. For sleep recordings, that is, slow-wave sleep (SWS) and rapid eye movement (REM) sleep, animals were placed in a central sleep box with bedding and access to *ad libitum* food and water.

### Recordings

Recordings were performed during the light phase of a 12 h day/night cycle following a postsurgery recovery period of no less than 8 d. Recordings were conducted using the DacqUSB acquisition system (Axona). Signals were amplified between 6,000× and 12,000× and bandpass filtered between 0.38 and 7 KHz for single-unit detection. Spike detection and unit sorting was performed based on tetrode configuration of electrodes to remove duplicates within octrode recordings; units that shared >5% common spike times were identified and the cluster with the lower spike rate was discorded. Additionally, units with a firing rate of <1 Hz were discarded. Local field potentials (LFPs) were sampled at a frequency of 4.8 kHz and downsampled to 2.4 KHz for all subsequent analyses. Automated spike sorting was performed using KlustaKwik (version 0.3.0; Kadir et al., 2014), and manual verification and adjustment was performed using Tint (Axona).

### Perfusion/histology

At the end of experiments, animals were given an overdose of sodium pentobarbital (60 mg/kg, Euthatal, Rhone Merieux) and transcardially perfused with 0.1 m PBS followed by 2.5% PFA in 0.1 m PBS. Brains were removed and postfixed overnight in 2.5% PFA before being transferred to 20% sucrose in 0.1 M PBS for ∼24 h. Sections were cut at 40 μm in the coronal plane using a cryostat. A 1-in-3 series was collected directly onto gelatin-coated slides and Nissl-stained (cresyl violet, Sigma-Aldrich) for verification of electrode location.

### Data analyses

All analyses were performed using custom scripts in MATLAB (version 2022a; The MathWorks) and Python 3.6.6, unless otherwise stated.

### LFP analyses

#### Power spectral density/coherence

A multitaper method (Bokil et al., 2010) was used for calculation of frequency domain spectral power, and the coherence between signals was estimated using magnitude-squared coherence. Power spectra were normalized to account for differences in electrode impedance by the integral of the power in the spectrum. Optimal LFP channels for use in analyses across a given session was chosen based on a combination of the spectral power in the theta band (4–12 Hz) during AWK recordings (MB and HPC), as well as the number of SWRs recorded during sleep recordings in the same recording day (HPC only).

#### Granger causality

In order to estimate the strength and directionality of information transfer between the medial MB, and the hippocampal LFP, we employed the MVGC toolbox, which utilizes the Wiener–Granger causality method (Barnett and Seth, 2014). This method assesses the degree to which the information content in one time domain signal predicts another in the frequency domain using autoregressive models. LFP from both brain regions were input as unfiltered signals, downsampled to a sampling frequency of 120 Hz. The model order was determined using the Levinson–Wiggins–Robinson algorithm and a maximum model order was defined as 50 sample points and the optimal order was defined using the Bayesian Information Criterion (BIC). Peak Granger causality values that were significant at an alpha of *p* < 0.001, as defined by Granger's *F* test, were compared in the theta band (6–12 Hz) across subjects.

#### Sleep detection (SWS/REM)

In the HPC, the awake state is characterized by high theta and low delta power. To detect episodes of SWS, the short time Fourier transformation of the signal was first calculated using the multitaper method (http://chronux.org; Mitra and Bokil, 2007) with a 10 s moving window and 4 s overlap. A theta/delta ratio for each time window was calculated using a peak detection algorithm on a smoothed (moving-average) of the maximum-normalized and squared signal of the spectrum. A global (whole recording) manual threshold based on visual classification was used to achieve optimal separation of AWK and NREM states (a lower than threshold TD ratio was classified as NREM and higher than threshold, AWK). Similarly, REM states were identified using the theta/delta ratio, with a speed threshold of 4 cm/s set to separate REM and AWK states.

#### SWR detection

Candidate SWRs were detected using peak detection threshold of the mean plus four standard deviations of the 150–250 Hz bandpass-filtered SWS-indexed hippocampal LFP signal, with a minimum interpeak time period of 200 ms. From ripple-triggered averages of candidate SWR times (20 ms), the number of oscillations above the standard deviation threshold were counted, and SWR events were considered valid if there were at least five oscillations above threshold within a 20 ms window surrounding the putative event.

#### REM–SWS comparisons

To assess changes in firing activity during different sleep states, REM and SWS epochs were first defined. REM events, identified as timepoints with high theta/delta power ratios and sub-threshold (<4 cm/s) movement speeds, were concatenated to define single REM epochs if there were fewer than 60 s between REM events. SWS epochs were defined similarly; SWS intervals were concatenated to form SWS epochs if there were fewer than 60 s between SWS events. For each day where there were identifiable REM epochs of over 30 s duration, the REM epoch with the longest duration and with neighboring SWS epochs was selected for analysis. Mean firing rates were calculated for MB and putative HPC pyramidal (PYR) cells during REM and the neighboring SWS epochs. Firing rate scores for each sleep state were calculated as follows:

$$\frac{\mathrm{sleep}\;\mathrm{state}\;\mathrm{mean}\;\mathrm{rate}-\mathrm{awake}\;\mathrm{mean}\;\mathrm{rate}}{\mathrm{sleep}\;\mathrm{state}\;\mathrm{mean}\;\mathrm{rate}+\mathrm{awake}\;\mathrm{mean}\;\mathrm{rate}}.$$

Units that did not fire in any of the sleep states were excluded from firing rate analysis.

Cell-pair correlations were calculated for MB units and HPC PYR units separately. Correlations were performed on single-unit spike trains binned into nonoverlapping 100 ms bins. Cells that were inactive in any of the three sleep states were excluded from analysis. Synchrony was defined as the mean within-region cell-pair correlation for each sleep state epoch. Due to a lack of normality of the distributions of firing rates and synchrony, we assessed whether there were differences between regions and sleep states using the nonparametric Friedman test, followed by Bonferroni’s corrected Dunn's pairwise post hoc tests.

### Single-unit analyses

#### Autocorrelation

Autocorrelation histograms were calculated as the cumulative sum of spikes found within ±0.5 s of each spike. Spike train autocorrelations were normalized so that the cumulative sum of 1 ms bins equal 1.

#### Running speed modulation

Running speed was derived from two-dimensional positional data (sampled at 10 Hz), using a ceiling-mounted infrared video camera, which in turn detected a small and a larger LED cluster mounted on the headstage. Positional information was resampled to 2.4 KHz and smoothed using a moving average filter across 100 ms windows. Spike rates were calculated for 1 cm/s bins (range 1–60 cm/s). Speed bins were included only if the range had been sampled for a minimum of 1 s within the recording session. Spike counts per cycle were calculated by first detecting the peaks of 4–12 Hz bandpass filtered hippocampal LFP. The integer rounded mean running speed of each theta cycle and the number of spikes for each corresponding cycle were calculated, allowing the mean average number of spikes per cycle in each speed bin (1–60 cm/s) to be determined. To assess the relationship between phase preference and running speed, we calculated the speed values for each spike by finding the spike triggered average of the running speed (200 ms windows). Spike phase values were calculated through piecewise cubic polynomial interpolation of phase angle values of the hippocampal LFP. Mean resultant vector (MRV) lengths (square root of the sum of the sin and cosine components of the phase values) of phase values from each valid speed bin were calculated. For each unit, the relationship between firing rate and running speed was assessed using linear regression. For units with a significant linear relationship between running speed and firing rate, we used weighted BIC scores (Wagenmakers and Farrell, 2004) to determine whether the fit could be improved with a sigmoid function (Spalla et al., 2022).

#### Waveform

Waveforms were extracted for each unit (50 bins of 0.04 ms) from the recording channel with the largest mean peak waveform amplitude. Waveforms were normalized by peak firing amplitude and any inverted waveforms were excluded from analysis. Putative hippocampal interneurons and pyramidal neurons were identified through KMeans clustering of the first two principal components of hippocampal unit mean normalized waveforms and the *z*-score of their mean firing rate (English et al., 2017; Nitzan et al., 2020). Putative interneurons were excluded from subsequent analysis. Waveform asymmetry was defined as the difference between the minimum amplitudes before and after the peak. Waveform peak–trough width was defined as the duration (ms) between the peak and the minimum amplitude after the peak. For sessions in which HPC and medial MB units were recorded concurrently, the peak–trough width and asymmetry of waveforms were compared between regions using Wilcoxon signed rank tests.

#### AHV

AHV was calculated as the first derivative of head direction (HD) based on the first 5 min of each central sleep box recording. AHV calculations were based on recordings of quiet wakefulness in the sleep box, rather than the circular track, to remove the potential confound of running speed-dependent changes in firing rate derived from changes in heading direction that naturally occur when running in a circle. First, HD was derived from the *x* and *y* coordinates of one small and one large LED cluster that were mounted on the headstage. HD data were resampled to 1 KHz and smoothed using a moving average filter across 100 ms windows. To obtain corresponding AHV values, a moving window (400 ms) surrounding each sample was indexed and the linear slopes (AHV values) were calculated. AHV values were assigned to 0.1 radians/s bins within a range of ±5 radians/s; bins with <1 s of occupancy were discarded. Firing rates within each bin were then calculated and tuning curves were generated for each unit. For each tuning curve, linear regressions were performed, separately, on clockwise (−5 to 0 radians/s) and counterclockwise (0–5 radians/s) head movements. A shuffling procedure was then performed, in which spike trains were circularly shifted (500 iterations) by randomly generated values between 20 and 100 s. Thresholds were then calculated from distributions of the estimates derived from linear regression of the shuffled-generated tuning curves. A unit was considered to be significantly AHV modulated if the estimate generated from nonshuffled data was greater than the upper bound (99% confidence interval) of the shuffle distribution (clockwise movement) or lower than the 1% confidence interval of the shuffle distribution for counterclockwise head movements.

#### Theta rhythmicity/phase

The theta index of a unit, as defined by the ratio of the power of the autocorrelogram within 1 Hz of the theta peak and the power within the entire spectrum of the autocorrelogram (Climer et al., 2015), provides a measure of the degree to which a unit engages in theta rhythmic firing. To assess theta cycle phase preference, a Hilbert transformation was applied to the 4–12 Hz bandpass filtered (3rd order Butterworth) hippocampal LFP. Spike phase values were calculated through piecewise cubic polynomial interpolation of the LFP instantaneous phase values. To classify units as phase entrained, phase tuning curves were generated from circularly shifted spike trains (1,000 iterations, shifted by a random value between −5 and 5 s). The MRV length was calculated for the shuffled and nonshuffled data and a unit was classified as significantly phase entrained if the nonshuffled MRV was greater than the 95th percentile of the distribution of MRVs based on shuffled spike trains.

#### Burstiness

The burst probability for each unit was calculated as the number of bursts divided by the sum of the number of bursts and spikes in the session. Burst detection was performed according to the method of Oosterhof and Oosterhof (2013). Bursts were defined as complexes with a minimum of three spikes, with a maximum interspike interval (ISI) to start a putative burst of 6 ms, and a maximum continuing ISI of 8 ms. Medial MB units were classified as dominantly bursting (DB) or sparsely bursting (SB) based on the methods of Simonnet and Brecht (2019). Specifically, principal component analyses were performed on the probability normalized log ISI histograms (LogISI; 0.001–10 s), and the first 20 ms from the central peak of the autocorrelograms for each unit. Ward's hierarchical clustering method was subsequently performed on the first principal component of the autocorrelation and the first to third principal components of the LogISIs. Principal component inclusion was based on the variance explained and the visual inspection of population LogISIs and autocorrelations when sorted by PCA score.

#### SWR-responsive activity

To assess the degree to which medial MB unit firing was positively, negatively, or unmodulated by hippocampal SWRs, spikes within ±500 ms of each SWR were summed to generate peri-SWR time histograms (PSTH) for each MB or HPC unit. To determine the significance of responsive firing, we generated PSTHs from shuffled datasets in which ripple event times were circularly shifted (1,000 iterations) by a random set value of between −5 and +5 s. An SWR responsiveness index (SWR RI) was calculated for each true PSTH by subtracting the mean firing rate from a 250 ms window around the shifted event time from the same period of each actual PSTHs. From each shuffled PSTH, the mean firing rate within the same window of the remaining 999 shuffled PSTHs were subtracted to generate a normal distribution of SWR responsiveness indices. The values of the SWR responsiveness indices of the nonshuffled PSTHs were compared with respect to the 5th and 95th percentiles of the shuffled distribution in order to assess the likelihood that the observed peri-SWR firing could happen by chance.

### Statistical analyses

Statistical analyses were performed either in R Statistics (version 4.3.1; provided in the public domain by the R Foundation for Statistical Computing; R Development Core Team, 2009, available at http://www.r-project.org/), in MATLAB (version 2022a; The MathWorks), or in Python (version 3.6.6—using the packages *pingouin* and *statsmodels*). For within unit analyses of firing rate correlates of movement, that is, running speed and AHV, linear and sigmoid models were fitted. For analyses in which parameters were compared between assigned groups, for example, DB versus SB, a random term (1|subject ID) was included in linear mixed effects models to account for the assignment of single units to different animal subjects. Given the difficulties associated with determining degrees of freedom in these instances (Bates et al., 2015), *p* values were obtained through likelihood ratio tests comparing nested models. Unless otherwise stated, the threshold for significance was set at *p* < 0.01. Graphs were generated using either MATLAB, the Python package *seaborn*, or the “ggplot2” package in R Statistics (Wickham, 2016), while figures were compiled in Inkscape (Inkscape version 0.92.4, The Inkscape Project, freely available at www.inkscape.org). Details of data distribution and statistical tests used can be found in Table 1.

## Results

**Figure 1.**
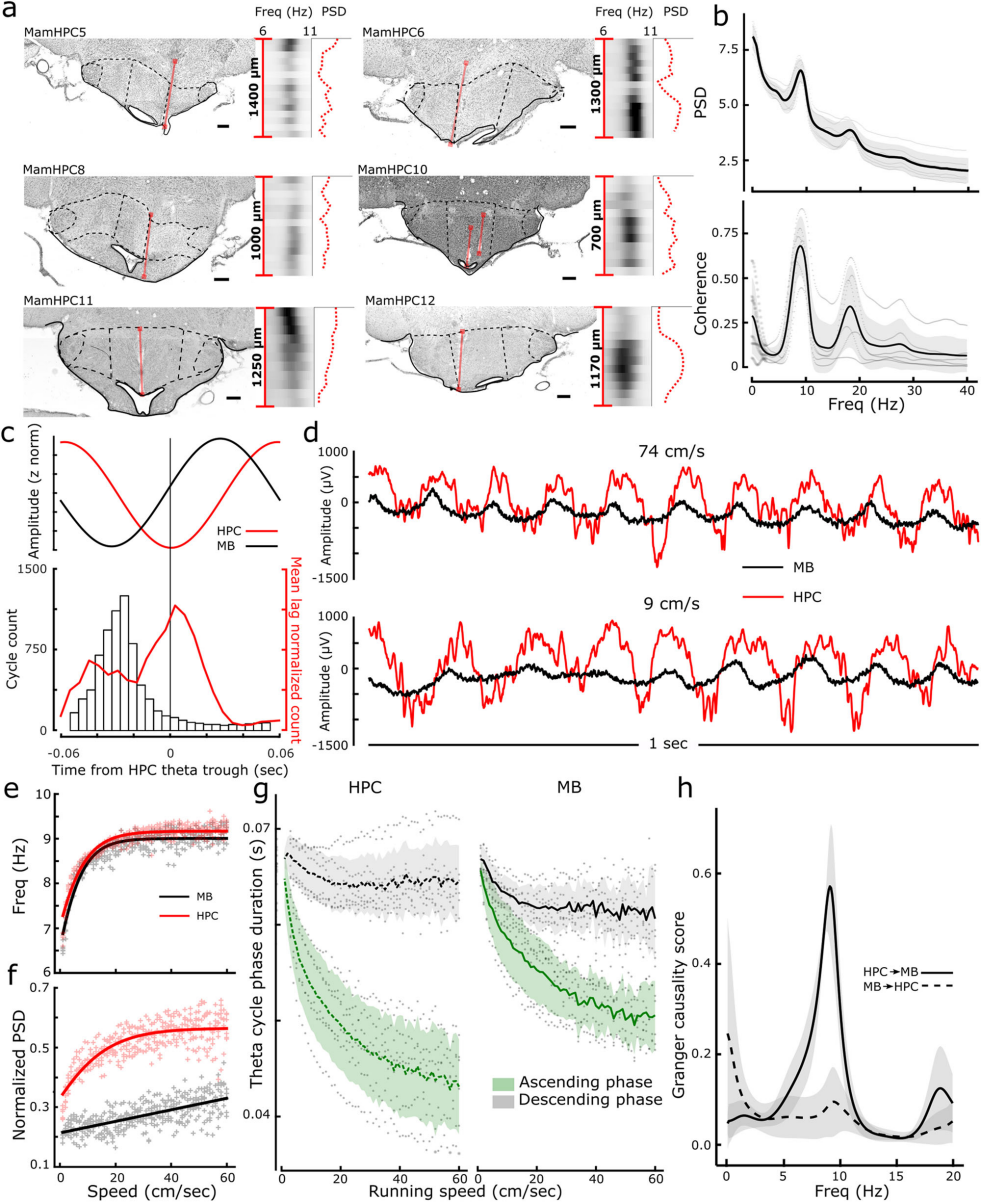
LFP characteristics of the medial MBs from conjoint hippocampal (HPC) and medial MB recordings. ***a***, Histological reconstructions of the MB electrode tracks (red lines). Black and white heatmaps show the theta band power of the LFP through the depth of the electrode track. Associated red dotted line shows the peak LFP power of the LFP versus electrode depth. ***b***, Normalized power spectral density (PSD; top) of the MB LFP showing peaks in the delta, theta, and first harmonic of theta and magnitude squared coherence between HPC and MB (bottom). ***c***, Mean phase lag in an example awake session between HPC (red) and medial MB (black) LFPs showing ∼30 ms lag (top) and a histogram of cycle lags within the session (bottom). Red trace shows the mean lag between the LFPs from all animals combined. ***e***, Example LFP traces, from conjoint MB and HPC recordings. Top traces, Mean running speed of 74 cm/s; bottom traces, 9 cm/s; illustrating change in frequency and cycle asymmetry. ***e***, Theta frequency of HPC and MB at different running speeds. Lines show the best fits for each region (both sigmoid functions). ***f***, Normalized power spectral density for medial MBs and HPC at different running speeds. Lines show the best fits of a sigmoid function for HPC and linear function for medial MBs. ***g***, Comparison of ascending (trough-to-peak) and descending (peak-to-trough) theta cycle durations with running speed. ***h***, Granger causality for HPC→MB and MB→HPC during active wakefulness. All scale bars in ***a*** are 250 µm.

Eight rats were implanted with octrodes and tetrodes targeting the medial MBs and the CA1 field of the HPC, respectively. Following histological assessment, six cases were included for subsequent analyses (Fig. 1*a*). In two cases (MamHPC10 and 11), the paths of the electrodes were located medially within pars medialis of the medial MBs. In the remaining four cases, the electrode paths (range, 700–1,400 µm paths; Fig 1*a*) were along or close to the pars medialis/pars lateralis boundary. Across all recording sessions (range, 10–14 recording days/rat), 543 single units were recorded from octrodes in the medial MBs while rats were running on a circular track. Of these, 293 units were recorded while rats were in the central arena, which included quiet wakefulness, SWS, and REM sleep (*n* = 145 units). Across the same recording sessions, 211 units were recorded from tetrodes implanted in the HPC (PYR, *n* = 179; INT, *n* = 32) during wakefulness, and 110 HPC PYR units were recorded during REM/SWS.

### LFP characteristics

LFPs are thought to represent the postsynaptic changes in membrane potential around the recording electrode. As the HPC (subiculum) provides a substantial input to the MBs, we first wanted to establish the extent to which the characteristics of the LFP recorded from the medial MB mirrored those recorded from CA1.

The power spectra of LFP of the electrodes placed in both CA1 and in the medial MBs had a dominant peak in the theta band (6–12 Hz), accompanying delta (1–4 Hz) peaks, as well as peaks in the beta (12–20 Hz) band, corresponding to the first harmonic of theta (Fig. 1*a*). During AWK periods, LFP signals from the two brain regions showed high peak magnitude-squared coherence in the theta frequency band (Fig. 1*b*; 6–12 Hz; 0.693 ± 0.154). It would be predicted that the unidirectional nature of the HPC→MB projection (Meibach and Siegel, 1977) would be reflected by a phase lag in theta oscillations between the HPC and medial MB signals. While the mean lag between the signals was consistent with this prediction (0.012 ± 0.006 s; Fig. 1*c*), there was considerably more variance both between subjects and within recording sessions than might have been expected. Theta frequency in the hippocampal and extrahippocampal limbic system increases as a function of running speed (Sławińska and Kasicki, 1998; Maurer et al., 2005; Jeewajee et al., 2008; Alex et al., 2016; Carpenter et al., 2017), thus we next looked at whether some of this variance in phase lag could be explained by differences in their relationship between phase lag and running speed. No relationship between phase lag and running speed was apparent (−1.361 ± 16.870; *χ*^2^_(1)_ = 0.0065; *p* = 0.9357^a^ ; details of all statistical tests can be found in Table 1)”), and, consistent with this finding, both regions showed a significant theta cycle symmetry-dependent association with speed (linear regression; HPC: *R*^2^ = 0.57, *p* < 0.0001^b^; MB: *R*^2^ = 0.52, *p* < 0.0001^c^), with this relationship best described by a saturating sigmoid function for both regions (sigmoid fit; HPC: *R*^2^ = 0.90, *p* < 0.0001^d^, sigmoid BICw = 1, linear BICw = 0; MB: *R*^2^ = 0.88, *p* < 0.0001^e^, sigmoid BICw = 1, linear BICw = 0; Fig. 1*d–g*). Similarly, there was a relationship between running speed and theta band PSD in each region (linear regression; HPC: *R*^2^ = 0.54, *p* < 0.0001^f^; MB: *R*^2^ = 0.45, *p* < 0.0001^g^), with the relationship for the HPC best described by a saturating sigmoid curve (sigmoid fit; HPC: *R*^2^ = 0.67, *p* < 0.0001^h^, sigmoid BICw = 1, linear BICw = 0) and the MB speed-power relationship best described by a linear fit (sigmoid fit; HPC: *R*^2^ = 0.90, *p* < 0.0001^i^, sigmoid BICw = 0.02, linear BICw = 0.98; Fig. 1*f*). While phase lag between signals did not present a clear picture of the direction of flow of information between HPC and medial MB, Granger causality analysis was more conclusive, with the magnitude of the theta band Granger causality score in the HPC→MB direction significantly higher than that in the inverse direction (0.48 ± 0.06; *t*_(10)_ = −8.2485; *p* = 9.0 × 10^−6j^; Fig. 1*h*).

### Medial MB unit characteristics

**Figure 2.**
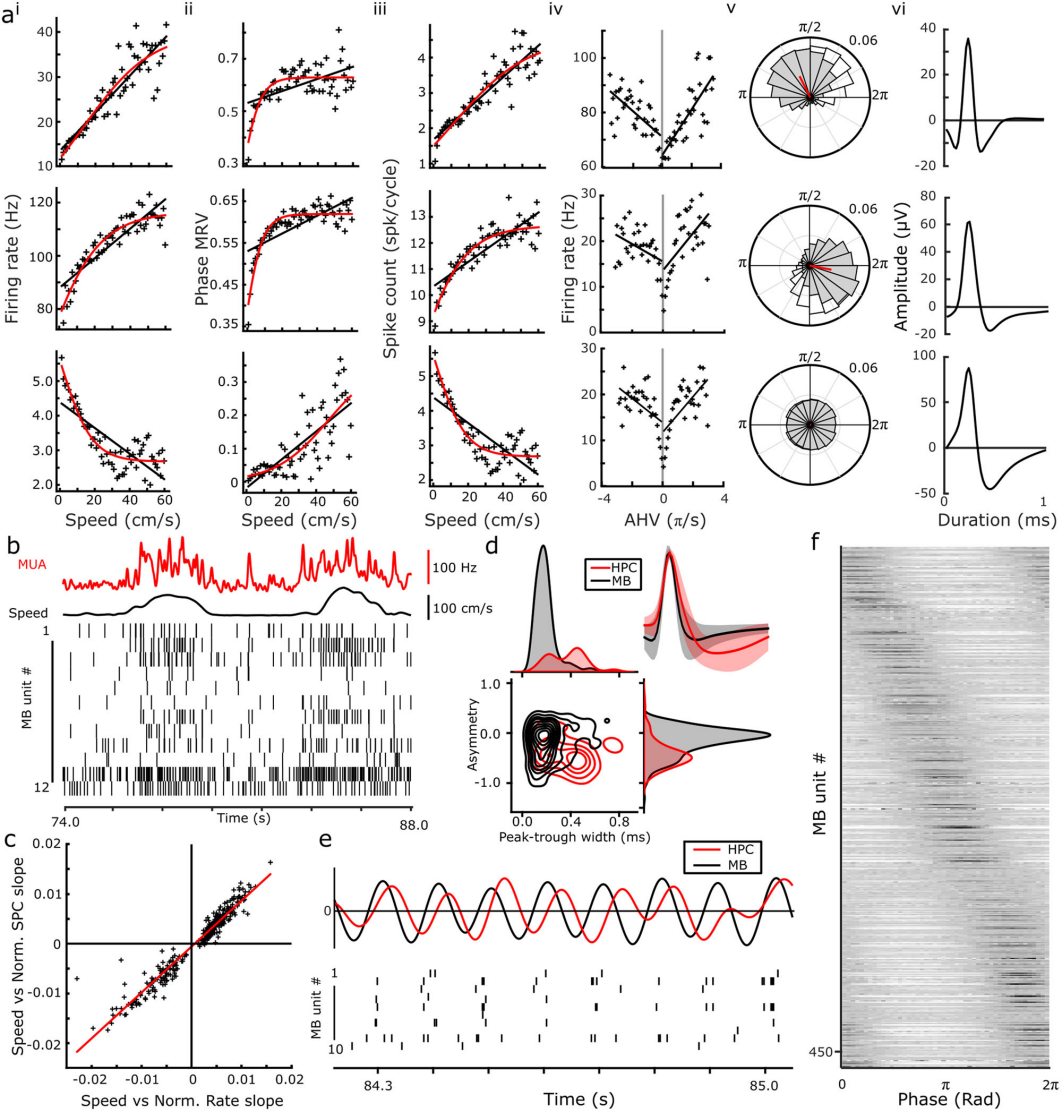
Physiological characteristics of MB units during awake exploration. ***a***, Firing characteristics of three representative medial MB units, showing speed-related changes in firing rate (i), theta phase MRV (ii), spikes per theta cycle (iii), AHV-related firing rate (iv), polar plot showing preferred theta phase (v), and spike waveforms (vi). Fits of linear and sigmoid functions are shown in black and red, respectively. Alongside speed/firing rate correlations, medial MB units also exhibited significant changes in strength of phase entrainment with running speed. ***b***, Example raster plots of synchronously recorded medial MB units showing population-dependent firing with respect to running speed. Red trace shows the combined multiunit activity (MUA) of the units depicted in the raster plots. ***c***, As demonstrated in the examples in ***a***, a unit's firing rate by running speed and its spikes per cycle by running speed relationship were highly correlated across the population of medial MB units recorded, reflecting an active increase in firing rate, that is, an increase in spikes/cycle. ***d***, Waveform asymmetry and peak–trough width of medial MB units (black) and hippocampal PYR units (red). Mean spike waveforms of medial MB (black) and hippocampal (red) units (top right). ***e***, Example epoch of synchronously recorded medial MB (black) and HPC (red) LFPs with corresponding raster plots of medial MB units exhibiting phase entrainment of firing during awake exploration. ***f***, Population phase preference of medial MB units (sorted by preferred phase), with each row representing the spike count phase histogram of a single medial MB unit.

The characteristics of medial MB neurons of rats in an awake state were found to be highly consistent with those reported in the work of Sharp and Turner-Williams (2005). The majority of units recorded fired either as a function of running speed and/or AHV and/or fired at a theta band frequency with phase entrainment to hippocampal theta oscillations (Fig. 2*a*). In addition, and consistent with studies in both slice (Llinas and Alonso, 1992) and anesthetized preparations (Kocsis and Vertes, 1994), we also found approximately a quarter of recorded medial MB units to exhibit complex bursting activity.

### Running speed-correlated firing

The firing rates of a large proportion of units (305/543 units; 56.2%) were found to be significantly correlated with running speed (Fig. 2*a*,*b*). By evaluating whether a sigmoid function improved the fit of models, units were classified as either linearly (*n* = 221/543; 40.7%) or nonlinearly correlated (*n* = 84/543; 15.5%). Of those 221 units that were linearly correlated with running speed 143 (64.7%) were positively correlated (Fig. 2*a*, top example). Of the 84 units that were best fit by a sigmoid function, 55 were positively correlated (65.5%; Fig. 2*a*, middle example), and 29 (34.5%) were negatively correlated (Fig. 2*a*, bottom example). As has been described in the medial entorhinal cortex (Hinman et al., 2016), a proportion of conjunctive speed correlated firing and spike phase entrained medial MB units (108/543; 19.9%) were found to have significant correlations between speed and the strength of phase entrainment, that is, the MRV length of medial MB spike phase histograms was correlated with running speed. To exclude the possibility that firing rate/running speed relationships were simply a function of phase-entrained units firing at a higher rate due to an increased number of theta cycles per second, we examined the relationship between running speed and spikes per theta cycle in medial MB units (Hinman et al., 2016). Consistent with an active increase in firing rate with running speed, regression analysis showed a highly significant positive relationship between running speed/firing rate and running speed/spikes per cycle (slope: 0.92 ± 0.01, *R*^2^ = 0.93, *p* < 0.0001^k^; Fig. 2*c*).

### AHV correlated firing

AHV tuning has been reported previously in the medial MBs (Sharp and Turner-Williams, 2005). To determine whether the firing rates of medial MB units varied as a function of AHV, we analyzed 5 min periods of quiet wakefulness in the central sleep box rather than the circular track which could have artificially affected lateral head movements. Linear regression performed on clockwise, and counterclockwise head movements independently, revealed that 23.2% (68/293) of medial MB units showed significant AHV tuning in both directions, while an additional 30.0% (88/293) of units showed asymmetrical tuning in either the clockwise (60/88 units) or counterclockwise (28/88 units) direction (Fig. 2*a*^iv^).

### Medial MB unit waveforms

The medial MBs comprise a population of excitatory neurons that predominantly project via collaterals to the anterior thalamic nuclei and Gudden's ventral tegmental nuclei (VTg; Van Der Kooy et al., 1978; Shen, 1983; Vann et al., 2007). We compared the waveform shapes of medial MB units with those of HPC PYR units, which have characteristically broad and asymmetric waveforms (Csicsvari et al., 1998). Both peak–trough width and waveform asymmetry differed between MB units and putative excitatory PYR. MB units were narrower (MB, 0.4 ± 0.01 ms; PYR, 0.74 ± 0.03 ms; *z*_(24)_ = 4.372; *p* < 0.001^l^) and more symmetrical (MB, −0.13 ± 0.02; PYR, −0.44 ± 0.03; *z*_(24)_ = 4.346; *p* < 0.001^m^) than PYR units (Fig. 2*d*).

### Theta phase entrainment

Nearly 50% (267/543) of medial MB units were found to fire rhythmically within the theta frequency band. In turn, we found that >85% (463/543) of units showed significant phase preference within theta (6–10 Hz) cycles (Fig. 2*a*^v^,*e*,*f*). Phase preference of medial MB units was distributed uniformly across the theta cycle both as a whole population, or when indexed based on conjunctive firing properties, for example, phase entrained, speed units (*n* = 543 medial MB units: Rayleigh *Z* = 0.47, *p* = 0.63^n^; Fig. 2*f*). Approximately 50% (272/543) of medial MB units were both phase entrained and responsive to running speed while nearly 26.7% (145/543) of medial MB units showed both theta rhythmicity and speed-dependent activity.

### Bursting

**Figure 3.**
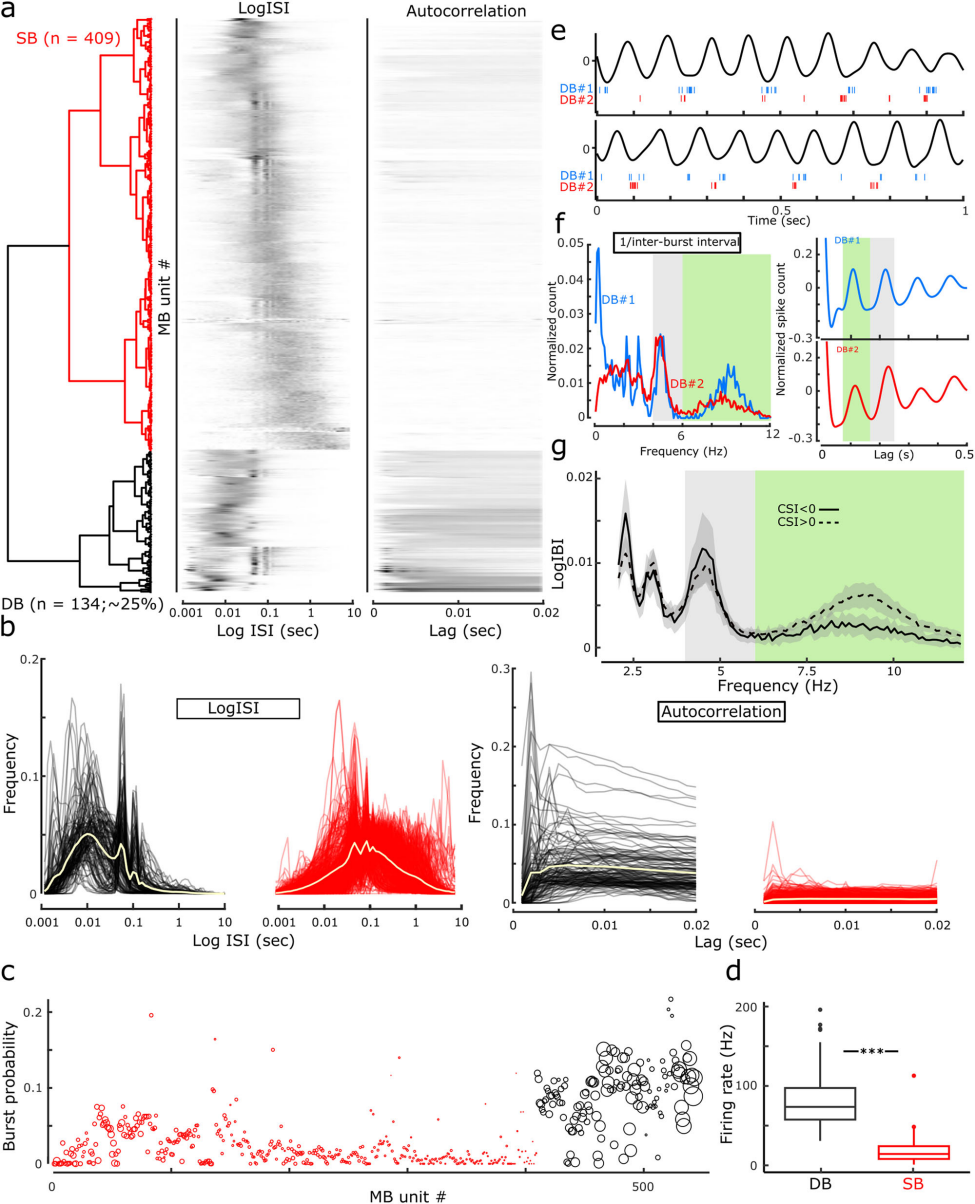
Bursting neurons in the medial MBs. Using the approach of Simonnet and Brecht (2019), PCAs 1–3 of the LogISI and the first PCA of the 0.02 s autocorrelation explained 80.1 and 87.0% of the variance, respectively. ***a***, Ward's agglomerative clustering identified two clusters (dendrogram) equivalent to the DB (*n* = 134; 24.7%; black) and SB (*n* = 409; 75.3%; red) of Simonnet and Brecht (2019); neighboring heatmaps show LogISI histograms (middle) and autocorrelations (maximum 0.02 s lag), ordered according to the clustering in ***a***. In each case a row corresponds to a single medial MB unit. ***b***, Left, Medial MB, DB units (black), had a higher mean peak in the LogISI ∼4–15 ms and a secondary peak shared by SB units (red) at ∼100 ms, corresponding to firing at a frequency within the theta band; Right, the mean 0.02 s autocorrelogram of DB units (black) showing a higher initial peak than SB units (red). The overlaid yellow trace in the four panels of ***b*** show the mean averages for each case. ***c***, A scatterplot showing the burst probability of medial MB units, sorted according to the hierarchical clustering procedure, is higher in DB classified units (black) in ***a***. Circle size represents unit mean firing rate. ***d***, Boxplot showing that as a population, firing rates in DB units are significantly higher than SB units. ***e***, Two example bursting medial MB units (DB#1, blue; DB#2; red) exhibiting cycle-skipping bursting (DB#1 in top example and DB#2 in the bottom trace), and nonskipping bursting activity (DB#2 in top trace and DB#1 in bottom example), at different points within the same recording. ***f***, Left, The corresponding log 1/interburst interval histograms and theta bandpass filtered 0.5 s autocorrelograms of example units DB#1 and 2. Peaks between 4 and 6 Hz correspond to cycle-skipping bursting activity, while the peaks at 6–12 Hz correspond to cycle-by-cycle bursting. Right, Autocorrelograms for both example units show theta rhythmicity, while those of DB#2 show a characteristic theta-skipping trace, likely to correspond to more dominant cycle-skipping burst activity. ***g***, Line plot showing the mean average LogIBI histogram of all medial MB units. Prominent peaks within the theta (6–12 Hz; green) and low theta (gray; 4–6 Hz) are highlighted.

An additional characteristic of AWK medial MB unit activity was the observation of bursting activity. We employed the approach of Simonnet and Brecht (2019) to aid the classification of bursting units. For this, we performed hierarchical clustering on PCA scores, calculated from the LogISI, as well as the 0.02 s lag of the autocorrelogram of all medial MB unit spike trains (Fig. 3*a*). Employing the same terminology as that used by Simonnet and Brecht in the subiculum, clustering of MB units identified two distinct groups: SB units (409/543 units; 75.3%) and DB units (134/543 units; 24.7%; Fig. 3*a*). Visual inspection of the LogISIs of DB and SB clusters (Fig. 3*b*) showed a LogISI peak in the DB cluster, spanning ∼2–15 ms that was largely absent in the SB cluster, reflecting the short latency between spikes within a burst event. Similarly, autocorrelograms of the classified groups reveal that DB units have high spike counts within a ∼5 ms lag (Fig. 3*b*), while SB classified units have a relative absence of spiking activity within a 0–10 ms lag. To further determine the effectiveness of this classification, a comparison of unit bursting probability between the clusters revealed that DB units had a significantly higher propensity to burst (difference in burst probability: −0.070 ± 0.003, *t* = −24.68, *χ*^2^_(1)_ = 408.84, *p* < 2.2 × 10^−16o^; Fig. 3*c*), while firing rates were also significantly higher in the DB group (difference between DB and SB: −20.941 ± 1.125, *t* = −18.62, *χ*^2^_(1)_ = 268.91, *p* < 2.2 × 10^−16p^; Fig. 3*d*).

Using this classification for DB units, we next assessed the degree to which bursting activity was associated with theta-dependent, running speed-dependent, and AHV-dependent unit firing. Subgroups of units exhibiting conjoint bursting/speed represented 74.6% (100/134) of DB units; DB/theta rhythmicity in spike trains represented 50.8% (68/134) of DB units, and 82.1% (55/67) DB units also fired as a function of AHV. Finally, units that exhibited combined bursting, speed-dependent firing, and theta rhythmicity accounted for 43.3% of the DB units.

### Theta cycle skipping

Inspection of medial MB unit autocorrelograms (0.5 ms bins) revealed characteristic examples of theta-skipping rhythmicity, wherein the secondary peak of the autocorrelogram (at ∼0.2–0.3 s lag) was larger than the primary peak (at ∼0.1–0.15 s lag; Climer et al., 2015). We next classified putative theta-skipping units by calculating the cycle skipping index (CSI; Kay et al., 2020), which defines the relative proportions of primary and secondary peaks of the autocorrelograms of theta rhythmic activity for each MB unit with a significant theta index. Of the 267 theta rhythmic medial MB units, 80 (30%) had a CSI greater than zero. Of these 80 putative theta-skipping medial MB units, 20 (25%) were also classified as DB units, which was representative of the proportion of DB units within the total medial MB population. To investigate the occurrence of theta cycle skipping DB unit firing, we calculated log interburst interval (LogIBI) histograms for all DB-classified medial MB units. Across all DB units, the mean LogIBI histogram had two dominant peaks, the first within the typical theta range (6–12 Hz) and a second at approximately half this frequency (4–6 Hz). Consistent with theta-skipping activity in DB units, the proportion of theta and half theta bursting LogIBI peaks were highly correlated with the CSI (6.13 ± 0.54; *t*_(68)_ = 11.42; *p* = 1.8 × 10^−17q^). Spike rasters for two theta-skipping DB units are shown alongside theta bandpassed HPC LFP (Fig. 3*e*,*f*); these examples indicate that individual medial MB units may exhibit theta-skipping and nonskipping activity at different timepoints during a single recording session.

We next compared the LogIBIs of theta-skipping DB units (CSI values >0) with nonskipping DB units. The integrated amplitude of the LogIBI in the theta band (6–12 Hz) of nonskipping (CSI < 0) DB units was significantly greater than that of theta-skipping DB units (nontheta-skipping vs theta-skipping units: 0.14 ± 0.05, *t* = 2.84, *χ*^2^_(1)_ = 7.64, *p* = 0.006^r^; Fig. 3*g*). Interestingly, however, nonskipping DB units did exhibit bursting in the 4–6 Hz (half-theta) band, indicating that while many DB units exhibited theta-skipping bursting activity, the relative proportion of theta nonskipping versus theta-skipping bursts is variable within the population (Fig. 3*f*).

### Physiological changes in the medial MBs during sleep

The rodent sleep cycle can be broadly split into AWK, SWS, and REM phases. Unlike AWK and REM phases, which are dominated by theta band activity, SWS is characterized by delta band (1–4 Hz) slow waves, which are highly synchronous across hippocampocortical regions. Similarly, the medial MB LFP is also characterized by delta band oscillations during SWS (medial MB, 1.25 ± 0.13 Hz; HPC, 1.57 ± 0.07 Hz).

**Figure 4.**
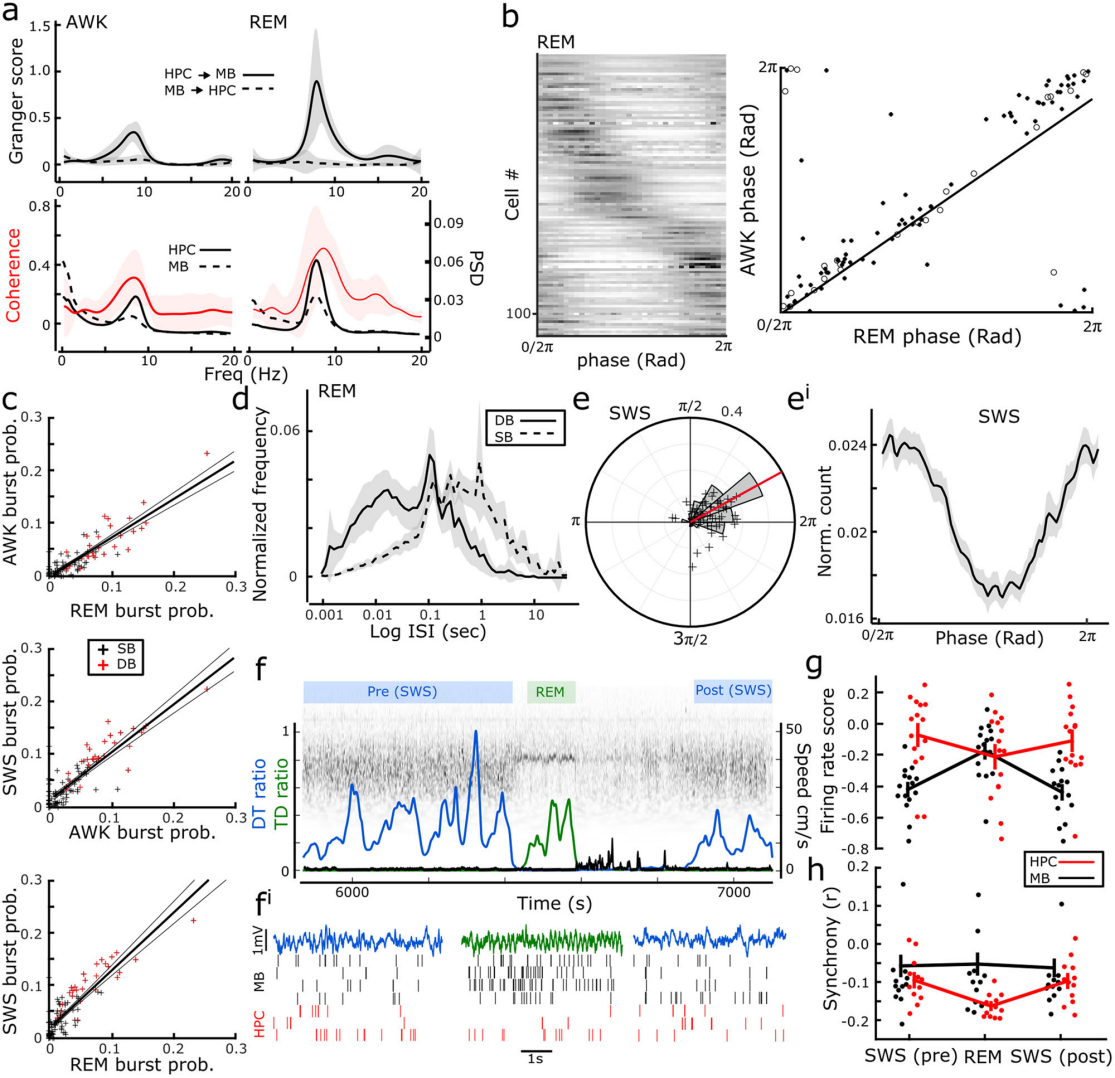
Characteristics of medial MB unit physiology across the sleep–wake cycle. ***a***, In AWK and REM, both theta-dominant states, Granger causality scores (within 6–12 Hz) were significantly higher in the HPC→MB direction than those in the reverse (top panels). Both HPC and medial MB LFPs had primary peaks in the theta band with significant coherence peaks at the corresponding frequency. ***b***, Units that were phase entrained during AWK maintained their preferred phase during REM. Solid points represent SB units and open circles, DB units. ***c***, Linear regressions of bursting probability of medial MB units across sleep–wake states. Medial MB units that exhibited complex bursting in AWK, also did so during SWS and REM. ***d***, Comparison of LogISIs of DB and SB units during REM showing that DB exhibited higher counts of spikes within <∼15 ms than SB units, reflecting DB units’ preserved propensity to burst during REM. ***e***, ***e*^i^**, Phase histogram with overlaid scatterplot of MRV length and preferred phase for each medial MB unit (***e***) alongside the mean normalized spike count phase histogram of the proportion of medial MB units (56%) that showed a phase entrainment to the downstate of 1–4 Hz oscillations during SWS (slow waves; ***e*^i^**). ***f***, Representative example of a wavelet-transformed epoch of REM sleep falling between two SWS epochs, and the physiological characteristics used for REM (high theta/delta ratio; green) and SWS (high delta/theta ratio; blue) detection, both with no movement speed; note the brief post-REM wakefulness (black line showing running speed). ***f*^i^**, Example 5 s epochs of wideband HPC LFP from REM (green) and neighboring SWS (blue) epochs with corresponding raster plots of synchronously recorded MB (black) and HPC PYR (red) single units. ***g***, AWK normalized firing rate scores of HPC (red) and medial MB (black) units within REM and neighboring SWS epochs. ***h***, Synchrony across sleep state transitions for HPC (red) and medial MB (black) units. Dots show individual points and error bars show mean ± SEM for each region at each sleep state.

As found during AWK periods, the multitaper power spectra of both the medial MB and hippocampal LFP during REM showed dominant peaks in the theta band (medial MB, 7.80 ± 0.28 Hz; HPC, 7.76 ± 0.25 Hz; Fig. 4*a*). The magnitude-squared coherence of HPC and medial MB signals was high in the theta frequency band during REM (coherence, 0.557 ± 0.056; peak coherence frequency, 8.203 ± 0.397 Hz; Fig. 4*a*). As found in AWK (Fig. 4*a*; also see Fig. 1*d*), the magnitude of theta band Granger causality scores was significantly higher in the HPC→MB than MB→HPC direction during REM (Granger score HPC→medial MB vs medial MB→HPC: 0.84 ± 0.17, *t*_(6)_ = 4.86, *p* = 0.003^s^; Fig. 4*a*).

Medial MB units that showed theta rhythmicity and/or theta cycle phase preference during AWK typically showed the same activity during REM. As a population, the preferred firing phase of medial MB units that exhibited significant theta phase preference in both states of arousal was highly correlated between these states (circular correlation coefficient = 0.88, *p* = 1.4 × 10^−7t^; Fig. 4*b*). Similarly, medial MB units that showed a propensity to burst during AWK were also highly likely to burst during both SWS and REM states (REM/AWK: 0.73 ± 0.04, *t*_(107)_ = 20.3, *p* = 1.7 × 10^−38u^; REM/SWS: 0.91 ± 0.05, *t*_(107)_ = 18.2, *p* = 1.2 × 10^−34v^, AWK/SWS; 1.1 ± 0.06, *t*_(107)_ = 18.4, *p* = 1.9 × 10^−35w^; Fig. 4*c*,*d*). Meanwhile, inspection of spike-triggered averages of medial MB units in SWS epochs showed delta-dominant spike-associated activity. Finally, phase spike count histograms of SWS spikes showed that a proportion of medial MB units were significantly phase entrained during SWS (61/108 medial MB units; 56%) and they fired almost exclusively during the downstate of slow waves (1–4 Hz; mean resultant phase, 28.8^o^, Rayleigh *Z* = 57.75, 7.4 × 10^−33x^; Fig. 4*e*,*e*^i^).

To investigate state-specific changes in activity during sleep, cell firing rates and synchrony were compared between HPC PYR and medial MB units during REM epochs (mean duration, 104.1 ± 2.7 s) and their neighboring SWS epochs (duration: SWS pre, 600.9 ± 29.9 s; SWS post, 673.6 ± 25.8 s; Fig. 4*f*). The SWS epochs that preceded and followed REM epochs were included in this analysis to assess whether any REM-related changes in firing rates or synchrony were reversed on re-entry into SWS, that is, not simply related to elapsed time. Medial MB units had firing rates of 4.49 Hz ± 0.91 and 4.14 Hz ± 0.7 in the pre- and post-SWS, respectively, while during the theta-dominant REM and AWK states, their firing rates were 7.03 Hz ± 1.1 and 8.95 Hz ± 1.18, respectively. Friedman tests revealed a difference between firing rate scores across sleep states and regions (15 sessions; *χ*^2^_(5)_ = 29.93; *p* < 0.001^y^; Fig. 4*g*). Post hoc pairwise Dunn tests revealed lower firing rate scores for MB units during SWS epochs than REM [SWS(pre)-REM: *z*_(14)_ = −2.13, *p* = 0.012^z^; SWS(post)-REM: *z*_(14)_ = −2.47, *p* = 0.002^aa^], and no difference between SWS epochs either side of REM [SWS(pre)-SWS(post): *z*_(14)_ = 0.33, *p* = 1^ab^]. Across all sleep states, MB firing rates were lower than during AWK states [SWS(pre): *z*_(14)_ = −3.41, *p* < 0.001^ac^; REM: *z*_(14)_ = −3.12, *p* < 0.001^ad^; SWS(post): *z*_(14)_ = −3.41, *p* < 0.001^ae^]. There was no difference between HPC firing rate scores between REM and SWS states [SWS(pre)-REM: *z*_(14)_ = 0.93, *p* = 1^af^; SWS(post)-REM: *z*_(14)_ = 0.27, *p* = 1^ag^; SWS(pre)-SWS(post): *z*_(14)_ = 0.67, *p* = 1^ah^]; however, HPC firing rate during REM was lower than AWK (*z*_(14)_ = −2.27, *p* = 0.023^ai^), while firing rates during both SWS states were no different than AWK [SWS(pre): *z*_(14)_ = −0.57, *p* = 0.57^aj^; SWS(post): *z*_(14)_ = −1.70, *p* = 0.09^ak^].

Synchrony (mean cell-pair correlations) was compared between regions and sleep states for all sessions with at least one cell pair in each region (*n* = 12). The Friedman tests revealed a difference in synchrony across sleep states and regions (*χ*^2^_(5)_ = 23.29; *p* < 0.001^al^; Fig. 4*h*). Post hoc pairwise Dunn tests found no change in synchrony in the medial MBs between sleep states [SWS(pre)-REM: *z*_(11)_ = −0.22, *p* = 1^am^; SWS(post)-REM: *z*_(11)_ = −0.23, *p* = 1^an^; SWS(pre)-SWS(post): *z*_(11)_ = −0.33, *p* = 1^ao^], while synchrony in HPC decreased in REM and recovered in the subsequent SWS epoch [SWS(pre)-REM: *z*_(11)_ = 4.15, *p* = 0.001^ap^; SWS(post)-REM: *z*_(11)_ = 3.82 *p* = 0.002^aq^; SWS(pre)-SWS(post): *z*_(11)_ = −0.33, *p* = 1^ar^]. Together, these comparisons reveal a dissociation of sleep state-related modulation of activity between regions, with MB units maintaining synchrony throughout all stages of sleep, despite changes in their firing rate, and HPC PYRs showing higher synchrony in SWS than in REM.

### Medial MB unit firing is associated with hippocampal SWRs

Three recent studies have implicated the medial MBs as an important target node in the propagation of hippocampal SWR activity by the subiculum. Firstly, Kitanishi et al. (2021) demonstrated that a considerable proportion of MB- but not ATN-projecting subiculum neurons fire as a function of hippocampal ripples; secondly, Kinnavane et al. (2018) showed that MB-projecting vesicular glutamate transporter 2 (VGLUT2) subiculum neurons send collateral projections to the retrosplenial cortex; thirdly, Nitzan et al. (2020) demonstrated that stimulation of these VGLUT2 subicular projection neurons elicits ripple-like activity in the retrosplenial cortex. Taken together, this would suggest that some neurons in the medial MBs may receive an SWR-related input. With this in mind, we next assessed whether medial MB unit activity changes with respect to HPC SWRs during SWS.

**Figure 5.**
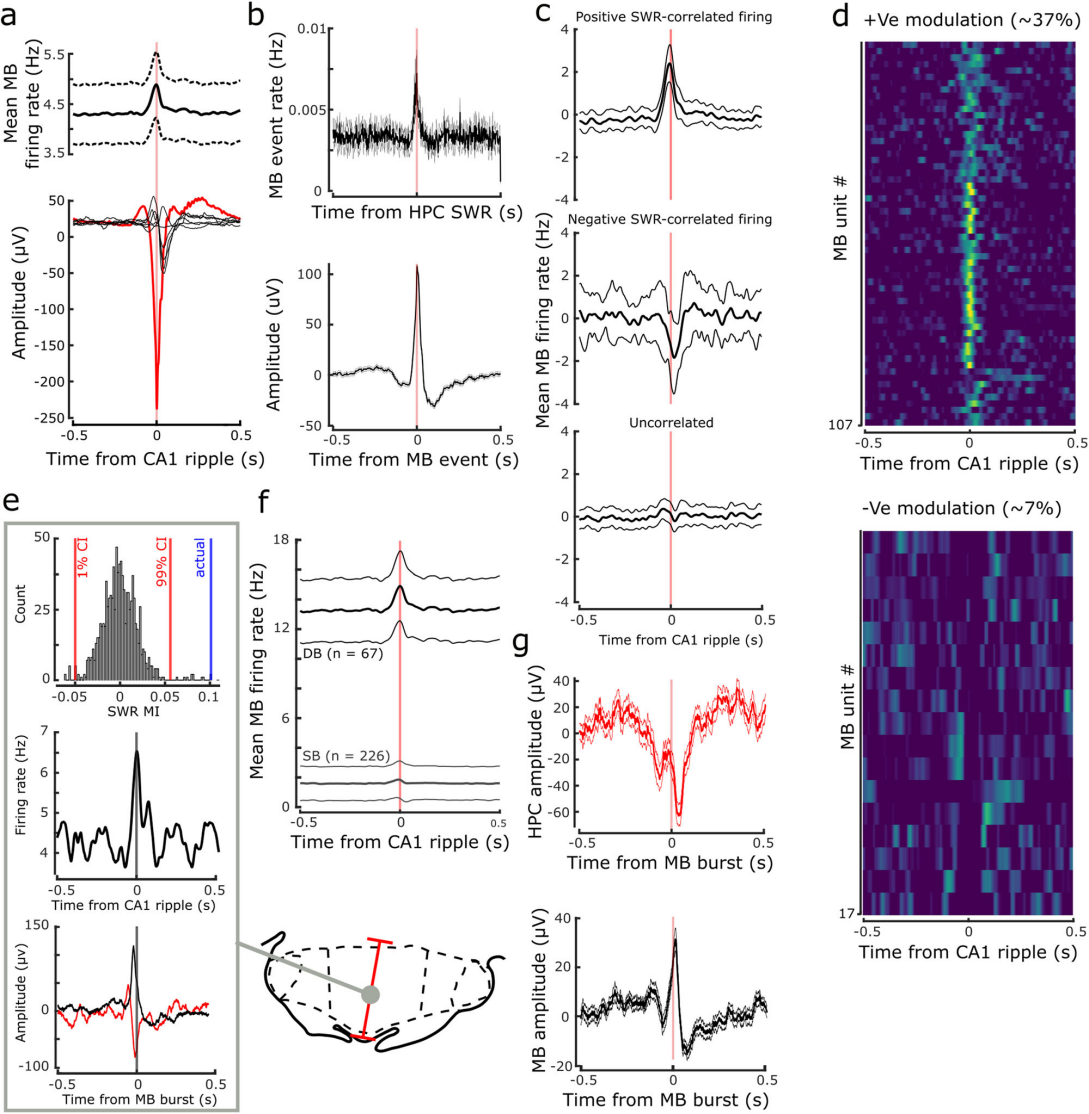
Hippocampal SWR-dependent firing in the medial MBs. ***a***, HPC ripple-triggered average of the HPC (red) and the MB (black) LFPs. Individual MB black traces (*n* = 6) represent the mean for each animal. Top panel shows the medial MB population-wide firing rate change with respect to HPC-detected ripples. ***b***, Peri-event time histogram showing the mean count of MB detected events with respect to putative SWRs detected in the hippocampal LFP (150–250 Hz; top); bottom, event-triggered average of high-frequency (100–250 Hz) events detected in the MB LFP. The resulting waveforms share the characteristics of putative MB response events observed by proximity to hippocampal SWRs (Fig. 5*a*) or through burst-triggered averages of SWR responsive firing units (Fig. 5*g*). ***c***, Mean firing rate of medial MB units in the time window surrounding detected hippocampal SWRs. Top, middle, and bottom panels include units with significant positive, negative, or uncorrelated firing, respectively. ***d***, Heatmaps with each row representing the convolved mean firing rate of a medial MB unit with respect to detected HPC SWRs. Top and bottom panels show the units (*n* = 107 and *n* = 21) with significant increases and decreases in firing rates, respectively. ***e***, Representative example of a medial MB unit that exhibited a significant increase in firing rate around the detected HPC ripple event times. The SWR responsiveness index (SWR RI) of the unit was higher than the 95% CI of a shuffle distribution of SWR RIs (see Materials and Methods for details). The example is also a DB unit. Burst-triggered averages (bottom) of the HPC (red) and MB (black) LFP showed typical HPC SWR and a medial MB response events. ***f***, Mean firing rate of DB versus SB medial MB units, with respect to HPC ripple events. Firing rates of both DB- and SB-classified units were significantly correlated with HPC ripple events; however, the mean magnitude of firing rate change was greater in the DB units. ***g***, Mean average burst-triggered average of the HPC (red) and MB (black) LFP during SWS revealing large amplitude events corresponding to putative HPC SWRs and medial MB response events.

Medial MB spike-triggered averages of the hippocampal LFP during SWS revealed, in many cases, putative SWR events. The same analysis applied to the medial MB LFP showed that putative hippocampal SWRs were often associated with corresponding low frequency events in the medial MB LFP. To address whether these were hippocampal SWRs and medial MB response events, we next detected HPC ripple events (150–250 Hz) in the hippocampal LFP. Ripple event-triggered averages of the hippocampal LFP provided validation of our ripple detection approach (Fig. 5*a*, red trace), with the mean average across animals showing a characteristic SWR shape. The same approach with the medial MB LFP showed that the mean response event waveform, that is, a putative medial MB response event, was remarkably consistent between animals. Temporally, the depolarization trough of the medial MB response events had a lag of ∼40 ms (lag from ripple detection, 0.042 ± 0.001 s; Fig. 5*a*) with respect to the HPC ripple event, which would be consistent with the events being temporally associated. To determine whether medial MB response events were temporally related to hippocampal SWRs, we next detected high gamma-ripple band (100–250 Hz) events in the medial MB LFP and generated peri-event time histograms of medial MB response events with respect to hippocampal SWRs. Counts of MB events within a 25 ms window around SWRs were significantly greater [threshold set at *p* < 0.01 when compared with shuffled (*n* = 1,000) ripple time distributions] in 20/32 recording sessions analyzed (Fig. 5*b*). Event-triggered averages of detected MB events appeared to be highly consistent in shape with those detected by both medial MB spike- and burst-triggered averages, as well as in hippocampal ripple-triggered averages of the medial MB LFP (Fig. 5*b*).

We next determined whether medial MB unit firing rates were correlated with ripple events. For this, we generated peri-event time histograms from which SWR modulation indices were calculated (Fig. 5*c–e*). To evaluate whether changes in MB unit occurred by chance, the SWR responsiveness index was compared with confidence intervals of a distribution of 999 SWR modulation indices generated from shuffle distributions based on 1,000 circularly time-shifted (0–5 s) ripple time series (see Materials and Methods for further detail). Of 293 medial MB units recorded during SWS, 44% (125/293) of medial MB units exhibited a significant increase (*n* = 106; Fig. 5*d*, top panel) or decrease (*n* = 19; Fig. 5*d*, bottom panel) in firing rate. Furthermore, when cross-referenced with the bursting (DB/SB) classification outlined in Figure 3, 13% (38/293) of medial MB units exhibited conjunctive bursting and SWR-dependent changes in firing rate. Indeed, as a population, medial MB units that exhibited SWR-dependent firing had a significantly higher propensity to burst than non-SWR-dependent units (burst probability: 0.024 ± 0.01, *t*_(284)_ = 3.50, *p* = 5.3 × 10^−4as^; Fig. 5*f*).

For further evidence that medial MB DB units were exhibiting SWR-dependent firing, we generated burst center-triggered averages of all DB units during SWS. Burst-triggered averages of hippocampal LFP showed event waveforms that were characteristic of hippocampal SWR events (Fig. 5*g*; see also Fig. 5*e* for representative example unit). Similarly, burst-triggered averages of medial MB LFP during SWS produced an event waveform that was characteristic of putative medial MB SWR response events.

## Discussion

**Figure 6.**
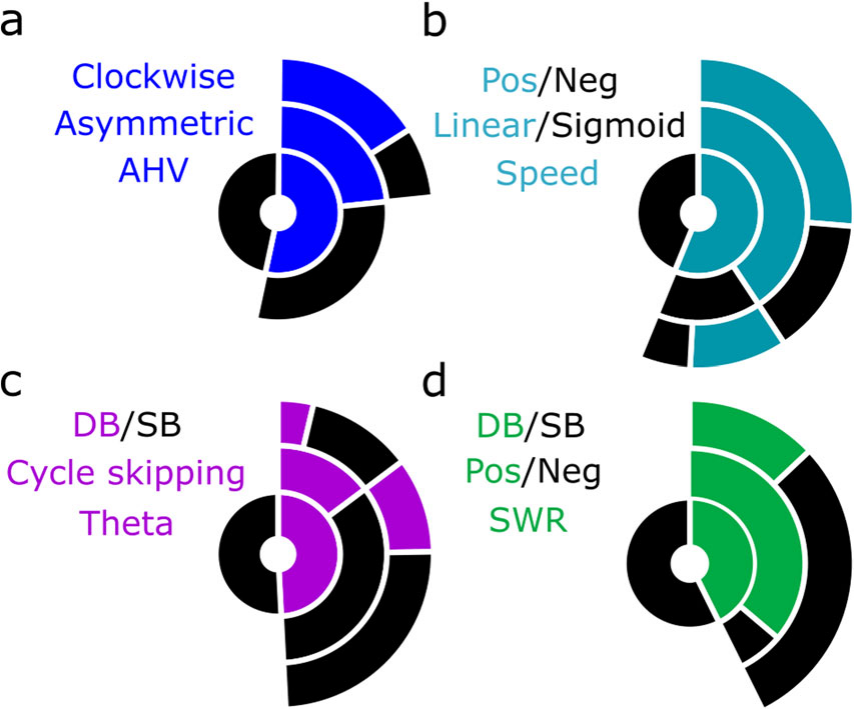
Summary charts of medial MB cell firing properties in wake and sleep. ***a–d***, Donut charts displaying the proportion of cells with firing rates related to the labeled property, further denoted by label color. Proportions of ***a***, AHV-dependent units (blue), separated by whether they are asymmetric (blue) or not (black), and the direction of movement by which asymmetric AHV units are modulated (clockwise, blue; counterclockwise, black) ***b***, Speed-dependent units, their best fitting polynomial (linear or sigmoid fit), and the direction of the relationship between speed and firing rate; ***c***, theta modulated units, the presence of theta cycle skipping, and the proportions of these units that are DB or not (SB); and ***d***, SWR-responsive units, the direction of their SWR-modulation, and the proportion of SWR units exhibiting bursting.

There are surprisingly few physiological studies focusing on the medial mammillary nuclei (medial MB), in contrast to the lateral mammillary nuclei, whose electrophysiological properties have been well studied with respect to their role within the HD system (Dillingham and Vann, 2019). In the present study, we classified over 500 medial MB units and found the physiological characteristics, and relative proportions of cells, to be in line with earlier reports (Sharp and Turner-Williams, 2005). However, in addition to cells showing running speed-related firing, AHV, theta firing rhythmicity, and theta phase preference, we report, for the first time in awake rodents, complex bursting activity and cycle skipping firing/bursting in medial MB spike trains. By recording from the medial MB across the sleep wake cycle, we found that across theta-dominant states (AWK/REM), theta-related characteristics, that is, phase preference and theta rhythmicity in spike trains of units, were largely conserved, and the bursting characteristics of cells was consistent across states, including SWS. Finally, we report that a proportion of medial MB units (37%) exhibit hippocampal SWR-responsive firing (Fig. 6).

**Figure 7.**
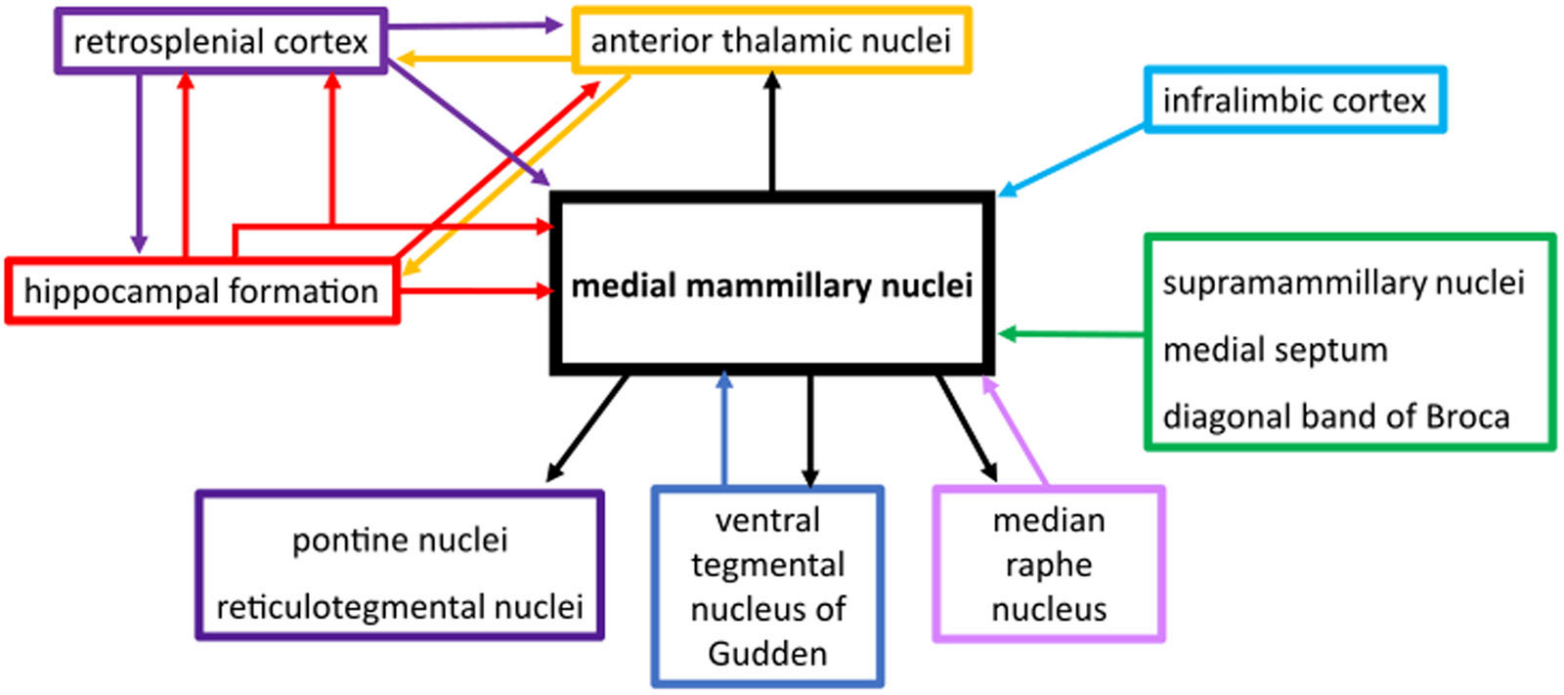
Schematic of the MBs’ principle efferents and afferents. The collateral projections from the hippocampal formation (subiculum) to the retrosplenial cortex and MBs are shown. The MB efferent projections arise from collateral fibers but these are not depicted here.

We found similar proportions of units with running speed-dependent firing (56.2%) to Sharp and Turner-Williams (2005;57.9%). However, we found fewer units to be positively correlated (65 vs ∼90%) and, while Sharp and Turner-Williams found speed-dependent firing in the medial MB to be linear, we found that the fit of 27.5% of units was significantly improved by fitting a sigmoid function. A nonlinear fit suggests that firing rates plateau at modest speeds in these units, similar to the saturating speed cells reported in the medial entorhinal cortex (Hinman et al., 2016). The discrepancy in findings is likely due to the speed correlations being assessed over a shorter range in the Sharp and Turner-Williams study (0–40 cm/s) making the saturation in firing rate less apparent. As a population, the running speed-responsive firing properties of medial MB units share a number of characteristics with those reported in hippocampal and parahippocampal regions (Hinman et al., 2016) and anterior thalamic nuclei (Lomi et al., 2023), that is, in proportions of units that show either linear or saturating speed relationships (Fig. 2*a*). It is currently unclear how the speed signal in the medial MBs contributes to the wider speed-dependent circuitry. The medial MBs might passively relay the speed signal from the hippocampal formation to the anterior thalamic nuclei; however, as the medial MBs also receive vestibular inputs via the ventral tegmental nucleus of Gudden (VTg; Irle et al., 1984), they may have a role in integrating movement-related information with theta frequency (Fig. 7).

Approximately one quarter of medial MB units (24.7%) were found to have DB characteristics (Fig. 6), and the propensity for DB neurons to burst was remarkably consistent across arousal states. The majority of these bursting units also conjointly fired in relation to speed, angular velocity, and/or theta. This bursting activity may be particularly important for the synchronization of activity within the medial MBs and may result in an enhanced, more reliable transmission of information to the anterior thalamic nuclei (Lisman, 1997; Csicsvari et al., 1998; Zeldenrust et al., 2018). Spike trains can also be segregated into single spike and bursting activity, facilitating the transfer of two different information streams (Oswald et al., 2004). The presence of such bursting activity in the medial MBs raises the question of whether the medial MBs might act to segregate spatial, motor, and theta-related information, transmitting this information to the anterior thalamic nuclei through parallel information streams, or whether this bursting activity acts to ensure the stability of transfer of particularly salient information during mnemonic processing.

The medial MBs receive input from theta-dominant structures, that is, the HPC (subiculum), the VTg, and septum (Fig. 7; Dillingham et al., 2021). Consistent with these inputs, we found a large proportion of medial MB units (∼50%) that exhibit rhythmic firing at theta-frequencies with a broad distribution of preferred theta phases, which aligns with previous findings (Kocsis and Vertes, 1997; Sharp and Turner-Williams, 2005). However, we also observed theta cycle-skipping firing in the medial MBs. While theta cycle-skipping unit activity has been reported in the HPC (Harris et al., 2003), parahippocampal cortices (Deshmukh et al., 2010), prefrontal cortices (Tang et al., 2021), and nucleus reuniens (Jankowski et al., 2014), it has not previously been reported in the medial MBs. Across these different regions, theta-skipping cells have been linked to the representation of different environments and different future trajectories (Jezek et al., 2011; Kay et al., 2020; Robinson and Brandon, 2021; Tang et al., 2021). The presence of theta cycle skipping units implicates the medial MBs as a node in this prospective coding network. Given the importance of the medial MBs for spatial working memory and the acquisition of spatial tasks (Santin et al., 1999; Vann and Aggleton, 2003), it will be important to assess how theta cycle skipping in the medial MBs contributes to these functions.

Our interest in physiological changes in the medial MBs across the sleep–wake cycle was driven primarily by recent, complementary findings on subicular–medial MB projections: (1) subicular–medial MB units show SWR-dependent firing (Kitanishi et al., 2021); (2) VGLUT2 subicular neurons send SWR information to the retrosplenial cortex (Nitzan et al., 2020); and (3) this VGLUT2 subicular–retrosplenial projection sends collaterals to the medial MBs (Kinnavane et al., 2018). Given the role of SWRs in the propagation of the hippocampal representation to downstream regions through highly coordinated replay events, and their implication in memory consolidation (Buzsáki, 2015), we sought to determine whether the medial MBs were in receipt of, and their activity modulated by, this hippocampal output. To do so, we first used SWR-triggered averages of LFP to determine whether the excitatory gain seen in hippocampal SWR events is reflected in the medial MB LFP (Fig. 5*a*,*b*); and second, we used SWR-triggered averages of MB unit spiking to determine whether this information may be further transferred to downstream targets of the medial MBs in the form of SWR modulated spiking and bursting activity (Fig. 5*c–g*). Our finding that a considerable proportion of medial MB units (∼37%) increased their firing rate around hippocampal SWR events supports the idea that subicular projections to the medial MBs carry SWR-related information during SWS. An interesting aspect of SWR-responsive firing in the medial MBs was the combined contribution of both DB and SB units, suggesting that the two primary efferent targets of the medial MBs, the anterior thalamus and VTg, may be receiving multiple SWR-related information streams. Ripple-correlated bursting activity within the medial MBs, rather than isolated spikes, might also suggest that SWR-responsive activity in the medial MBs is modifying rather than simply relaying hippocampal information. As well as showing that nearly all medial MB-projecting units from the subiculum exhibit strongly SWR-responsive firing, Kitanishi et al. (2021) showed that anteroventral thalamic nucleus-projecting subiculum neurons showed reduced, weak, or non-SWR-responsive firing. Anterior thalamic neurons do, however, show SWR-coupled activity (Viejo and Peyrache, 2020); it is therefore a possibility that propagation of hippocampal SWR information to the anterior thalamus is dependent on the subiculum–medial MB pathway. Indeed, the medial MBs, which receive at least some of the same SWR-related input as the retrosplenial cortex, are well placed to influence the temporal coupling of hippocampal SWRs, thalamocortical spindles, and delta waves in the cortex. The nesting of these events during SWS is associated with improved consolidation of hippocampal-dependent memory (Maingret et al., 2016; Kim et al., 2019), and induction of spindles through optogenetic stimulation of the thalamic reticular nucleus (with which anterior thalamic nuclei share connectivity; Lozsádi, 1995) improves hippocampal-dependent consolidation by increasing frequency of coupling of these events in the cortex (Latchoumane et al., 2017). Like the medial MB, another major hippocampal output target, the lateral septum (LS), contains a high number of SWR-responsive neurons (Wirtshafter and Wilson, 2019; Tingley and Buzsaki, 2020; Howe and Blair, 2022). In the medial MBs, high-frequency (100–250 Hz) SWR response events were temporally correlated with hippocampal SWRs (Fig. 5*a*); in the LS, high-frequency events were often synchronized with SWRs but were also observed independently of HPC, and stimulation of GABAergic LS neurons elicited high-frequency events locally (Tingley and Buzsaki, 2020). Unlike the LS, medial MB neurons are uniformly excitatory but in the absence of GABAergic neurons locally within the medial MBs, high-frequency oscillations could feasibly be generated through recruitment of GABAergic VTg neurons via mammillothalamic axon collaterals (Hayakawa and Zyo, 1989; Umaba et al., 2021). In addition to medial MB LFP response events closely lagging behind HPC SWRs (∼40 ms), medial MB unit firing rates exhibited changes prior to the peak power of HPC SWRs (Fig. 5*c*), further indicating a close temporal relationship between HPC and medial MB high-frequency events during sleep. These findings may warrant future exploration of the precise temporal relationship between the two regions during SWRs, as well as a further exploration of the potential presence of VTg-medial MB generated high-frequency events.

A second reason for assessing sleep cycle-dependent changes was that firing rates in VTg have been reported to increase dramatically during REM (Bassant and Poindessous-Jazat, 2001) compared with that during wakefulness. Given that the medial MBs have dense reciprocal connections with VTg, we were interested to see if medial MB unit firing showed similar characteristics. Our observations of firing rate changes across the sleep cycle found the contrary. Medial MB units exhibited a small but significant reduction in firing rates during REM sleep when compared with AWK. This reduction in firing rate may reflect increased inhibition from GABAergic VTg inputs (Bassant and Poindessous-Jazat, 2001). Alternatively, given that a large proportion of medial MB units exhibit motion-responsive firing (e.g., running speed and AHV), it is possible that it reflects the absence of vestibular and motion-related input during sleep. However, we also reported a greater reduction in MB unit firing rates during SWS sleep than that during REM sleep; thus reduction in motility alone cannot explain the extent of the changes in MB firing rates across sleep states. The modulation of MB firing rates across sleep states is in stark contrast to the relative stability of HPC PYR rates. There were also differences between medial MBs and PYR units in terms of sleep state-related synchrony: medial MB units maintained synchrony across sleep state transitions while PYR ensembles desynchronized during REM. Taken together with our findings that medial MB units maintain their theta phase preference during REM and exhibit phase entrainment to the downstate of slow waves during SWS (Fig. 4*e*). It is possible that MB units are entrained to the dominant oscillatory frequency across sleep states and changes in firing rates across sleep reflect those frequencies that define REM and SWS. When considering that a significant proportion of MB units exhibit SWR-mediated increases in firing, the overall reduction of medial MB firing rates during SWS may provide a mechanism that reduces background activity in order to amplify SWR events to the ATN.

The present study extends our understanding of the function of the medial MBs by identifying novel mechanisms through which they support memory processing. The majority of medial MB neurons exhibit conjugate firing properties, for example, a combined running speed, AHV, bursting, and theta rhythmicity representation, suggesting that unlike the parallel lateral MB pathway, in which the degree of complexity in neuron representations increases from the bottom-up (Dillingham and Vann, 2019), the medial MBs are involved in higher-order processing. Further functional heterogeneity is demonstrated by the varied responses of medial MB units to hippocampal SWR events, as well as widespread theta cycle-skipping activity. These combined properties are consistent with the medial MBs playing an active role in the formation of complex spatial memories (McNaughton and Vann, 2022; Milczarek et al., 2024).

## Synthesis

Reviewing Editor: Christie Fowler, University of California Irvine Decisions are customarily a result of the Reviewing Editor and the peer reviewers coming together and discussing their recommendations until a consensus is reached. When revisions are invited, a fact-based synthesis statement explaining their decision and outlining what is needed to prepare a revision will be listed below. The following reviewer(s) agreed to reveal their identity: NONE.

Two experts have reviewed this manuscript, and both felt the findings greatly expand on previous electrophysiological characterizations of the medial mamillary body nucleus. Among the novel characterizations include reporting of complex bursting patterns and theta cycle skipping. Several major and minor issues were noted, as described below in the synthesis, which will need to be addressed for reconsideration. In addition, full statistical reporting is required in a table format per eNeuro policy, and the manuscript needs to be formatted consistent with eNeuro author guidelines.

In this manuscript, Dillingham et al. conducted a thorough electrophysiological profiling of the medial mamillary body region, with concurrent recording of the CA1 region of the hippocampus for comparison. The authors recorded over 500 medial MB units and found their electrophysiological characteristics with respect to animal speed, angular head momentum, as well as theta entrainment and phase preference largely corroborate findings of Sharp and Turner-Williams (2005). In addition to this validation, the authors report novel findings in the following ways: 1) they found complex bursting activity (divided into dominant and sparsely bursting clusters) and theta cycle skipping in medial MB units, 2) they recorded across various sleep-wake states and found medial MB units conserve rhythmicity and phase preference across the awake (AWK) and rapid-eye movement (REM) stages, and maintain similar bursting propensity across all arousal states, 3) a portion of medial MB units are strongly associated with hippocampal sharp wave ripple event (SRPs). Overall weaknesses relate to difficulty with readability, particularly of the results section, and figure formatting.

Major comments:

1) The writing would benefit from more concise writing and clarity. To illustrate, the following sentence from the manuscript is difficult to comprehend for the reader: "As one would expect, given the abundance of theta rhythmicity and theta phase- entrainment in medial MB unit firing, medial MB spike - and burst-triggered averages of the HPC LFP during AWK and REM - showed medial MB spike-related epochs of high amplitude theta oscillatory activity."

2) The order of the figures as they are presented in the text are different from the order presented in each figure. This makes the paper quite difficult to follow.

3) Given the extensive description of anatomical and functional connectivity in the discussion section., it may be helpful to include one final figure representative illustration on the known afferents and efferents to and from the MB, such as the anterior thalamic nuclei, ventral tegmental nuclei, subiculum/RSP, that may inform readers on the physical anatomy involved in the temporal coupling events reported.

4) In the sleep-state analysis, the justification for dividing SWS epochs into a Pre and Post REM sleep epoch is missing. What does this additional division add?

5) Figure 5A, top panel shows positively correlated medial MB firing activity centered to SWR events. It seems that increased firing (peak of the black line) actually shortly precedes the SWR event (red line). In contrast, negatively correlated units (middle panel) seem to respond shortly after the SWR event. This observation is not discussed and more broadly the precise timing of the medial MB response in relation to SWR events is not really explored by the authors.

6) Many figure captions are insufficient. For example, for figure 1E there are 4 subfigures in the subpanel and only the top left described.

7) Line 6 - 10 in Page 17, because the author emphasized that running speed influenced the both the firing properties and the theta oscillations in MB, it is better to inform the readers whether the Granger causality in the HPC-MB direction is dependent on running speed.

8) In Figure 2A, does quadratic fitting work better than exponential fitting? If yes, please explain why the firing rate, phase MRV and spike count decreased when the running speed is higher than 40 cm/s. If not, please use exponential fitting.

9) Figure 4G and relative text, reporting firing rate score can make the comparison between MB and HPC neurons easier. However, for the readers' information, please also report the mean firing rate of MB neurons during SWS, REM and AWK relatively.

Minor comments:

1) The authors state: "In turn, we found that more than 85% (463/543) of units showed significant phase preference within theta (6-10 Hz) cycles (Fig 2A, E)." Uncertain how figure 2A shows phase preference.

2) Data for figure 6 may be more clearly represented as a Venn diagram

3) There are a number of general aesthetic improvements that could be made:

a. Overall font sizes used in the axes and titles of all figures are too small.

b. Ambiguously labelled units for both the x- or y- axes across many figures:

i. 1C placement of the speed on the y-axis is odd, as this is not the unit of measurement, which is typically indicated in parentheses. Placing the speeds horizontally above the traces as a title is clearer.

ii. 4B (REM phase (rad), should just be phase (rad)). The term REM phase may refer the entire REM epoch so this is confusing.

iii. Figure 3B. The x-axis should say log ISI, not ISI (sec).

iv. Units would helpful on graph subticks, e.g. 1E-1F, 4A.

4) Figure 1E, the left panels are confusing, the grey lines are both solid and dotted. I guess the grey lines are not for HPC, and the coherence is between two regions, so the figure legend in the upper right panel doesn't work for the left panels. Please put figure legend for the left two panels.

5) Line 18 in Page 16, a left parenthesis is missing.

6) Figure 2B, please put scale bar or y axis for MUA and speed to indicate the value.

7) For easier comparison with literatures, please use time instead of number of bins to define peak-trough width of waveform.

8) Please find a better font for pi in the figures, now it looks like "n" (perhaps due to the poor resolution of the draft pdf file).

9) Figure 4Fi, please add voltage scale bar for the raw LFP.

10) Figure 2A shows that cell 1 and 2 have phase preference in the polar bar plot. Because this figure has so many subpanels, one might not even realize the polar bar plot is part of Figure 2A. It would be of benefit to given the figure sub-captions and refer to the polar bar plot only.

## Author Response

Dear Professor Fowler, Thank you for your positive feedback on our manuscript. Your comments, and those of the Reviewers, were extremely thoughtful and have helped us improve the manuscript. We have addressed each of the comments in turn and highlighted the changes in the manuscript.

Major comments:

1) The writing would benefit from more concise writing and clarity. To illustrate, the following sentence from the manuscript is difficult to comprehend for the reader: "As one would expect, given the abundance of theta rhythmicity and theta phase- entrainment in medial MB unit firing, medial MB spike - and burst-triggered averages of the HPC LFP during AWK and REM - showed medial MB spike-related epochs of high amplitude theta oscillatory activity." Thank you for this comment. We have re-written parts of the manuscript to improve clarity.

2) The order of the figures as they are presented in the text are different from the order presented in each figure. This makes the paper quite difficult to follow.

We have re-ordered the figures so that they are aligned to the flow of the manuscript and easier to follow.

3) Given the extensive description of anatomical and functional connectivity in the discussion section., it may be helpful to include one final figure representative illustration on the known afferents and efferents to and from the MB, such as the anterior thalamic nuclei, ventral tegmental nuclei, subiculum/RSP, that may inform readers on the physical anatomy involved in the temporal coupling events reported.

We have now provided a schematic of the mammillary bodies' efferents and afferents as requested (Figure 7).

4) In the sleep-state analysis, the justification for dividing SWS epochs into a Pre and Post REM sleep epoch is missing. What does this additional division add? The pre-post REM SWS was provided separately for a couple of reasons. The rationale was to ask whether the rates and synchrony are state related, i.e., if they change from SWS to REM is that change then reversed on re-entry into SWS? Without this, you could argue that the changes in rates and synchrony might just be related to duration of sleep i.e., the longer the period of sleep the lower the rates. Practically speaking, REM epochs are shorter and there was rarely more than 1 REM epoch in a sleep session, so it was not feasible to look at REM-SWS-REM.

We have now added the following text to provide a clearer rationale: "SWS epochs that preceded and followed REM epochs were included in this analysis to assess whether any REM-related changes in firing rates or synchrony were reversed on re-entry into SWS, i.e., not simply related to elapsed time." 5) Figure 5A, top panel shows positively correlated medial MB firing activity centered to SWR events. It seems that increased firing (peak of the black line) actually shortly precedes the SWR event (red line). In contrast, negatively correlated units (middle panel) seem to respond shortly after the SWR event. This observation is not discussed and more broadly the precise timing of the medial MB response in relation to SWR events is not really explored by the authors.

Thank you very much for this question relating to Figure 5A (note: this figure has now been moved to 5C). In this analysis the spike triggered averages are based upon the point of the hippocampal SWR event with the highest power. However, SWR events can be of variable length, with the point of peak power occurring away from the centre of the SWR. Ideally, one might wish to use the start point of an SWR for this analysis, however the identification of start (and end) points of SWR events also proves tricky - requiring arbitrary thresholding that may lead to mis-identification of the actual start points.

Because we use the peak power of the hippocampal SWR, the MBs will be receiving SWR-related information prior to, and after, the labelled event time (0s, red line). This is reflected in the apparent ramping up and down (or vice versa for negatively correlated cells) in the ~150 ms around the peak hippocampal SWR time. Nevertheless, it is interesting that the peaks of these events seem to fall just before or just after, the peak power of hippocampal SWR - and is worthy of more comment and discussion. However, for a variety of technical reasons, discussion of this needs to be caveated. First, the difficulty in defining precise start and end points of SWR events, alongside the very small apparent offsets of peaks for the SWR-correlated units, makes it hard to be confident that this observation reflects the reality of MB responses to hippocampal SWR. In addition to this, the timing and power of SWR events varies depending on where exactly SWRs are recorded from hippocampus - further increasing the difficulty of determining precise temporal relationships of SWR-related events. Finally, our analysis of medial MB LFP response events related to HPC SWRs revealed a medial MB LFP lag of ~40ms (Fig. 5a), reducing the likelihood that SWR activity in HPC is typically preceded by an increase of activity in MBs.

Ideally, to determine whether MB activity can precede HPC-SWR activity, a suite of new experiments would be required. This might entail probe recording along the transverse axis of the hippocampus, alongside MB recordings; or the use of optogenetic approaches to elicit, or inhibit, hippocampal ripples during concurrent recording of MBs. However, we believe a deeper exploration of this observation falls outside of both the remit and the technical limits of the recording methods used in this current manuscript.

We have now explored this in the discussion:

In addition to medial MB LFP response events closely lagging behind HPC SWRs (~40 ms), medial MB unit firing rates exhibited changes prior to the peak power of HPC SWRs (Fig. 5C), further indicating a close temporal relationship between HPC and medial MB high-frequency events during sleep. These findings may warrant future exploration of the precise temporal relationship between the two regions during SWRs, as well as a further exploration of the potential presence of VTg-medial MB generated high frequency events.

6) Many figure captions are insufficient. For example, for figure 1E there are 4 subfigures in the subpanel and only the top left described.

Thank you - we have increased the detail in the figure legends to better describe the subpanels, and where necessary have split up subpanels, i.e. Fig 1E is now split into 1B and 1E.

7) Line 6 - 10 in Page 17, because the author emphasized that running speed influenced the both the firing properties and the theta oscillations in MB, it is better to inform the readers whether the Granger causality in the HPC-MB direction is dependent on running speed.

Thank you for this - we have expanded our analysis to look at changes in Granger causality scores at different speeds. We determined the integral of Granger causality score within the theta band (6-12Hz) at speeds between 1 and 20cm/s.

We found that HPC-MB Granger causality scores increase with increasing speed (Response Fig 1. Left) - this fit was best described by a saturating sigmoid curve, similar to those we see for the relationship between speed and PSD in the theta band, and speed and theta frequency. This raised the question, what is the relationship between power in the theta band and Granger causality score? We found that with increasing theta power in the HPC (max. normalized for each day of recording), the Granger scores increase in a linear fashion (Response Fig 1. Center).

Because of this relationship between PSD and Granger score, we reassessed the speed and Granger score relationship after weighting the Granger scores by the normalized theta band power at each speed. Once this was accounted for, we found that there was no relationship between running speed and Granger score (Respond Fig 1. Right). In other words, it is possible that any changes we see in the strength of directionality of signal from HPC to MB occurs as a function of increasing power. This relationship between speed, power and Granger score may need to be further disentangled in future studies.

Response Fig1. Speed and granger causality. Left: Granger scores increase as a function of speed. This is best fit by a saturating sigmoid curve (red line). Middle: There is a linear relationship (black line) between hippocampal PSD at the theta band and granger score. Right: There is no relationship between speed and the PSD-weighted granger scores. Grey lines, each animal's mean granger score at different speeds. Blue line and shaded area, mean and sem. Black line - linear fit.

8) In Figure 2A, does quadratic fitting work better than exponential fitting? If yes, please explain why the firing rate, phase MRV and spike count decreased when the running speed is higher than 40 cm/s. If not, please use exponential fitting.

Thank you for raising this. We have replaced our use of a quadratic function with a sigmoid function. The fitting of this functional form has recently been used by Spalla, Treves & Bocarra (2022) to identify parahippocampal speed cells. We determined whether units were linearly or non-linearly tuned by comparing the weighted BIC scores for the fits of the linear and sigmoid functions (Wagenmakers and Farrell, 2004). This sigmoid function, which produces saturating curves, may also be more physiologically relevant than our previous use of quadratic terms.

The total number of speed-tuned units, and the relative proportions of linearly and non-linearly tuned units have changed (somewhat trivially) using this analysis. The broad picture that speed-tuned neurons are predominantly linearly- and positively-tuned remains consistent with the initial manuscript. Any changes in numbers of speed-tuned neurons have been reflected in subsequent analysis calculating the theta- and burst-conjunctivity of speed tuned neurons, and the outputs of these analyses have also been updated accordingly. The changes in numbers of speed tuned neurons have also been reflected in Figures 2 and 6.

9) Figure 4G and relative text, reporting firing rate score can make the comparison between MB and HPC neurons easier. However, for the readers' information, please also report the mean firing rate of MB neurons during SWS, REM and AWK relatively.

To improve clarity, we have now additionally reported the mean firing rates during each of the sleep epochs, as below: "Medial MB units had firing rates of 4.49Hz {plus minus} 0.91 and 4.14Hz {plus minus} 0.7 in the pre- and post-SWS, respectively, while during the theta-dominant REM and AWK states their firing rates were 7.03Hz {plus minus} 1.1 and 8.95Hz {plus minus} 1.18, respectively" Minor comments:

1) The authors state: "In turn, we found that more than 85% (463/543) of units showed significant phase preference within theta (6-10 Hz) cycles (Fig 2A, E)." Uncertain how figure 2A shows phase preference.

Thank you - while Fig 2AV shows examples of units with preferred theta phases Figures 2E and F better represent this phase preference throughout the population. We have modified the text accordingly to point readers to Fig 2E and 2F as well.

2) Data for figure 6 may be more clearly represented as a Venn diagram Thank you for this suggestion. We originally thought a Venn diagram would be a good approach but when we tried to present the data this way, we struggled to show the data clearly. There were a number of concentric circles and it all got a bit messy. We also tried a pie chart but again we just couldn't get it to clearly show the data. We found the donut charts, while still not perfect, to provide the best visual representation of the data and relative proportions of cells.

3) There are a number of general aesthetic improvements that could be made: a. Overall font sizes used in the axes and titles of all figures are too small. b. Ambiguously labelled units for both the x- or y- axes across many figures: i. 1C placement of the speed on the y-axis is odd, as this is not the unit of measurement, which is typically indicated in parentheses. Placing the speeds horizontally above the traces as a title is clearer ii. 4B (REM phase (rad), should just be phase (rad)). The term REM phase may refer the entire REM epoch so this is confusing. iii. Figure 3B. The x-axis should say log ISI, not ISI (sec). iv. Units would helpful on graph subticks, e.g. 1E-1F, 4A.

Thank you - we have amended all figures to generally improve aesthetics and clarity. We have increased font sizes and line widths, as well as improving unit and axis labelling on figures as suggested.

4) Figure 1E, the left panels are confusing, the grey lines are both solid and dotted. I guess the grey lines are not for HPC, and the coherence is between two regions, so the figure legend in the upper right panel doesn't work for the left panels. Please put figure legend for the left two panels.

Thank you - we have rearranged this figure and updated the legend accordingly. This figure now better reflects the flow of the manuscript.

5) Line 18 in Page 16, a left parenthesis is missing.

Thank you for spotting this, we have now added the parenthesis.

6) Figure 2B, please put scale bar or y axis for MUA and speed to indicate the value.

Thank you - we have now added scale bars to this figure.

7) For easier comparison with literatures, please use time instead of number of bins to define peak-trough width of waveform.

This has now been updated in the figure and in the methods section.

8) Please find a better font for pi in the figures, now it looks like "n" (perhaps due to the poor resolution of the draft pdf file).

This has been fixed.

9) Figure 4Fi, please add voltage scale bar for the raw LFP.

Thank you - this has now been added.

10) Figure 2A shows that cell 1 and 2 have phase preference in the polar bar plot. Because this figure has so many subpanels, one might not even realize the polar bar plot is part of Figure 2A. It would be of benefit to given the figure sub-captions and refer to the polar bar plot only.

Subpanel labels have been provided in Figure 2A and legend has been changed accordingly.
